# Phytochemistry and Pharmacological Activities of *Rehmannia glutinosa*: A Comprehensive Review

**DOI:** 10.3390/molecules31193435

**Published:** 2026-09-27

**Authors:** Hyunkyung Kwak, Hwaryeong Lee, Isoo Youn, Eun Kyoung Seo

**Affiliations:** 1 College of Pharmacy, Ewha Womans University, Seoul 03760, Republic of Korea; hkkwak@ewha.ac.kr (H.K.); jongsky119@naver.com (H.L.); 2 Department of Biohealth-Convergence, Seoul Women’s University, Seoul 01797, Republic of Korea

**Keywords:** *Rehmannia glutinosa*, Orobanchaceae, phytochemistry, pharmacology

## Abstract

*Rehmannia glutinosa* (Orobanchaceae) has traditionally been used to control the level of blood sugar, stimulate tonification, and regenerate blood and fluid in the body. The roots can be processed by steaming, which alters the chemical profiles of the plant and helps to utilize it in various diseases. Although several review articles on *R. glutinosa* have been published, few have comprehensively summarized the chemical constituents isolated from this plant. Therefore, this review provides a comprehensive overview of the phytochemical components identified in *R. glutinosa*, which are categorized in several classes including iridoids, ionones, phenylethanoids, phenylpropanoids, flavonoids, lignans, terpenoids, alkaloids, and saccharides, together with their reported biological activities. The reviewed studies have demonstrated diverse biological activities, including antidiabetic, anti-obesity, anti-inflammatory, neuroprotective, organ-protective, cytotoxic, antioxidant, and antimicrobial activities. However, the available evidence is predominantly derived from in vitro and in vivo rodent models, and clinical evidence remains limited. Therefore, further studies are required to establish appropriate preparation methods and dosages, as well as evaluate the pharmacokinetic and pharmacodynamic profiles of *R. glutinosa* for its therapeutic applications.

## 1. Introduction

The genus *Rehmannia* Libosch. ex Fisch. & C. A. Mey. belongs to the Orobanchaceae family and is composed of seven species: *R. chingii* H.L.Li, *R. chrysantha* M.H.Li & C.H.Zhang, *R. glutinosa* (Gaertn.) Libosch. ex DC., *R. henryi* N.E.Br., *R. japonica* (Thunb.) Makino, *R. piasezkii* Maxim., and *R. solanifolia* P.C.Tsoong & T.L.Chin. [1,2]. *R. glutinosa* (commonly known as Dihuang) is one of the widely used medicinal herbs in Asian countries including Korea, China, and Japan. According to the “Catalogue of Ancient Classical Prescriptions” published by the State Drug Administration and the State Administration of Traditional Chinese Medicine in China in 2018, 22 out of 100 prescriptions included *R. glutinosa* [3].

In traditional medicine, the roots of *R. glutinosa* are processed into different forms for distinct medicinal applications. The dried form of *R. glutinosa* is characterized as having a “cold nature” and is traditionally used to clear heat, cool the blood, and treat constipation [3]. In contrast, prepared (steamed) *R. glutinosa* has a “warm nature” and is used to tonify yin and treat conditions such as irregular menstruation and premature graying hair [4]. Dramatic chemical changes occur during the steaming of *R. glutinosa*: the disappearance of iridoid glycosides, increased sweetness resulting from the conversion of oligosaccharides to monosaccharides, and the formation of 5-hydroxymethylfurfural through the Maillard reaction [5]. From the perspective of modern medicine, the roots of *R. glutinosa* and their constituents have been reported to show antioxidant, anti-inflammatory, antitumor, antidiabetic, organ-protective, neuroprotective, antidepressant, and immunomodulatory effects [6].

Although several research groups have published review articles on the phytochemical and pharmacological aspects of *R. glutinosa* [3,7], a comprehensive account of its constituents and their pharmacological activities has not yet been reported. In this study, more than 310 chemical constituents reported in the literature from 1982 to 2025 were comprehensively reviewed together with their biological activities. A literature search was performed on relevant publications retrieved from online databases (PubMed, SciFinder, and Google scholar), and the literature search covered studies published from 1982 to 2025 (the final search was conducted on 5 January 2026). The search strategy was conducted using the following keywords: “Rehmannia glutinosa”, “Rehmannia”, “phytochemistry”, “biological activity”, “pharmacology”, “review”, and “isolation”. Publications in journals with specific impact factors that underwent the peer-review process were considered for this review, and articles published in English were primarily considered. Original research articles reporting compounds isolated or characterized from *R. glutinosa* and/or their biological activities were considered eligible for inclusion.

## 2. Phytochemistry of *R. glutinosa*

Phytochemical investigations of *R. glutinosa* have resulted in the identification of various compounds, including iridoids and their glycosides [8,9,10,11,12,13,14,15,16,17,18,19,20,21,22,23,24,25,26,27,28,29], ionones and their glycosides [13,14,17,18,21,27,30,31,32,33,34,35,36,37,38], phenylethanoid glycosides [8,23,27,35,39,40,41,42,43,44], phenylpropanoids and their glycosides [3,35], flavonoids [8], lignans [18,33,35], terpenoids [18,19,31,35,37,38,39,41,45,46,47,48], alkaloids [10,29,49], and saccharides [10,40,50,51]. The class and name of the identified compounds from *R. glutinosa* are presented in Table 1.

### 2.1. Iridoids and Their Glycosides

The name “iridoid” originated from iridomyrmecin, which is a defensive chemical from the *Iridomyrmex* ants [52]. Iridoid is a cyclopentan-[*c*]-pyran monoterpenoid, composed with a cyclopentane ring fused to a six-membered oxygen-containing heterocycle. Most of the iridoids occur as glycosides in nature, with some as free or bis-compound forms [53]. There are also seco types of iridoids, in which the pyran ring is open. As the main active chemical constituents found in the roots of *R. glutinosa*, they include well-known key molecules such as aucubin, catalpol, and ajugol.

Aucubin (**1**) is a bicyclic iridoid glucoside with a [4.3.0] nonane skeleton, which contains 10 carbon atoms and lacks C-11. It is usually found in both *Rehmannia* and *Plantago* species [54,55]. When the plant is damaged, aucubin (**1**) undergoes enzymatic cleavage by *β*-glucosidase and causes browning through protein cross-linking as part of the plant defense response [56]. Genipin (**10**), geniposide (**11**), and geniposidic acid (**9**) share the same iridoid skeleton. Geniposide (**11**) is a glycosylated form of genipin (**10**), for both of which a methyl ester moiety exists at C-1. On the other hand, geniposidic acid (**9**) loses a methyl ester from geniposide (**11**) at the C-1 position. Genipin (**10**) works as a promising alternative for cross-linking agents, as it can fabricate hydrogels in drug delivery systems and tissue engineering [57].

**Table 1:** Phytochemical constituents isolated from *R. glutinosa.*

Compounds	Identification Status	Plant Part	Processing State	Identification Method	References
**Iridoids and their glycosides**					
aucubin (**1**)	reference matching	leaves and roots	air-dried	LC-MS	[8]
	isolated	roots and aerial parts	fresh	NMR	[9]
linaride (**2**)	isolated	leaves	air-dried	HPLC, NMR, MS	[8]
monomelittoside (**3**)	isolated	roots	dried	NMR, MS	[10]
melittoside (**4**)	isolated	roots	fresh	NMR	[9]
rehmaglutoside L (**5**)	isolated	roots	dried	NMR, MS	[10]
	isolated	roots	air-dried	NMR, MS	[11]
rehmannioside D (**6**)	isolated	roots	fresh	NMR	[9]
rehmaglutoside M (**7**)	isolated	roots	air-dried	NMR, MS	[11]
rehmaglutoside N (**8**)	isolated	roots	air-dried	NMR, MS	[11]
geniposidic acid (**9**)	isolated	roots	fresh	NMR, MS	[18]
	isolated	roots	dried	NMR	[21]
genipin (**10**)	isolated	roots	fresh	NMR, MS	[18]
geniposide (**11**)	isolated	roots	fresh	NMR	[16]
1-*O*-*α*-L-rhamnopyranosyl(1→6)-*β*-D-glucopyranoside (**12**)	isolated	roots	fresh	NMR	[17]
ajugol (=leonuride, **13**)	reference matching	leaves and roots	air-dried	LC-MS	[8]
	isolated	roots and aerial parts	fresh	NMR	[9]
	isolated	roots	air-dried	NMR, MS	[11]
	isolated	roots	-	NMR	[12]
ajugoside (**14**)	isolated	rhizomes	dried	NMR, MS	[13]
rehmannioside C (**15**)	isolated	roots	fresh	NMR	[9]
rehmaglutoside O (**16**)	isolated	roots	air-dried	NMR, MS	[11]
6-*O*-(*E*)-caffeoyl ajugol (**17**)	isolated	roots	dried	NMR, MS	[14]
rehmaglutoside A (**18**)	isolated	roots	air-dried	NMR, MS	[22]
6-*O*-*p*-coumaroyl ajugol (**19**)	isolated	roots	-	NMR, MS	[12]
6-*O*-(*E*)-feruloyl ajugol (**20**)	isolated	roots	-	NMR, MS	[12]
6-*O*-(*Z*)-feruloyl ajugol (**21**)	isolated	roots	-	NMR, MS	[12]
6-*O*-*p*-hydroxybenzoyl ajugol (**22**)	isolated	leaves	air-dried	HPLC, NMR, MS	[8]
	isolated	roots	-	NMR, MS	[12]
6-*O*-vanilloyl ajugol (**23**)	isolated	roots	-	NMR, MS	[12]
rehmaglutoside B (**24**)	isolated	roots	air-dried	NMR, MS	[22]
6-*O*-(4″-*O*-*α*-L-rhamnopyranosyl)vanilloyl ajugol (**25**)	isolated	roots	-	NMR, MS	[12]
rehmaglutoside C (**26**)	isolated	roots	air-dried	NMR, MS	[22]
7-deoxygardoside (**27**)	isolated	roots	fresh	NMR	[17]
8-epiloganic acid (**28**)	isolated	roots	-	NMR	[12]
gardoside (**29**)	isolated	roots	fresh	NMR, MS	[18]
feruloyl gardoside (**30**)	isolated	roots	fresh	NMR, MS	[18]
(8*S*)-7,8-dihydrogeniposide (=adoxoside, **31**)	isolated	roots	dried	NMR, MS	[10]
11-methylforsythide (**32**)	isolated	roots	fresh	NMR	[17]
mussaenoside (**33**)	isolated	roots	fresh	NMR	[19]
jioglutoside B (**34**)	isolated	roots	dried	NMR, MS	[14]
8-epiloganin (**35**)	isolated	roots	fresh	NMR	[19]
rehmaglutin H (**36**)	isolated	roots	fresh	NMR, MS	[18]
gardoside methyl ester (**37**)	isolated	roots	fresh	NMR	[19]
dihydrocornin (**38**)	isolated	roots	dried	NMR	[20]
rehmaglutoside E (**39**)	isolated	roots	air-dried	NMR, MS	[22]
rehmannioside G (**40**)	isolated	roots	dried	NMR, MS	[23]
rehmannioside H (**41**)	isolated	roots	dried	NMR, MS	[23]
dihydrocatalpol (**42**)	annotated through KEGG compound database	root-derived cell cultures	cultured cells	UPLC-MS^2^	[58]
catalpol (**43**)	reference matching	leaves and roots	air-dried	LC-MS	[8]
	isolated	roots and aerial parts	fresh	NMR	[9]
	reference matching	roots	fresh/dried/ steamed	HPLC	[59]
jioglutoside A (**44**)	isolated	roots	fresh	NMR, MS	[16]
rehmannioside A (**45**)	isolated	roots	fresh	NMR	[9]
rehmannioside B (**46**)	isolated	roots	fresh	NMR	[9]
rehmaglutoside J (**47**)	isolated	roots	air-dried	NMR, MS	[22]
rehmaglutoside K (**48**)	isolated	roots	air-dried	NMR, MS	[22]
jioglutin A (**49**)	isolated	roots	steamed	NMR, MS	[24]
jioglutin B (**50**)	isolated	roots	steamed	NMR, MS	[24]
jioglutin C (**51**)	isolated	roots	steamed	NMR, MS	[24]
rehmaglutin A (**52**)	isolated	roots	dried	NMR, MS	[25]
	isolated	roots	dried	NMR, MS	[26]
rehmaglutin B (**53**)	isolated	roots	dried	NMR, MS	[25]
	isolated	roots	dried	NMR, MS	[26]
rehmaglutin D (**54**)	isolated	rhizomes	dried	NMR, MS	[13]
	isolated	roots	steamed	NMR, MS	[24]
	isolated	roots	dried	NMR, MS	[25]
	isolated	roots	dried	NMR, MS	[26]
rehmannia A (**55**)	isolated	root tubers	-	NMR, MS	[27]
glutinoside (**56**)	isolated	roots	steamed	NMR, MS	[24]
	isolated	roots	dried	NMR, MS	[28]
6*β*-hydroxy-2-oxabicyclo [4.3.0]Δ^8,9^-nonen-1-one (**57**)	isolated	roots	fresh	NMR	[19]
jioglutolide (**58**)	isolated	roots	steamed	NMR, MS	[24]
jiofuran (**59**)	isolated	roots	steamed	NMR, MS	[24]
rehmaglutin C (**60**)	isolated	roots	dried	NMR, MS	[20]
	isolated	roots	dried	NMR, MS	[28]
rehmaglutin E (**61**)	isolated	leaves	air-dried	NMR, MS	[29]
rehmaglutoside D (**62**)	isolated	roots	air-dried	NMR, MS	[22]
**Ionones and their glycosides**					
aeginetic acid (**63**)	isolated	roots	dried	NMR	[60]
	isolated	roots	air-dried	NMR	[31]
frehmaglutin B (**64**)	isolated	roots	air-dried	NMR, MS	[31]
frehmaglutin B_1_ (**65**)	isolated	roots	fresh	NMR, MS	[61]
aeginetic acid 5-*O*-*β*-D-quinovoside (**66**)	isolated	roots	dried	NMR, MS	[47]
frehmaglutin F (**67**)	isolated	roots	fresh	NMR, MS	[62]
3-hydroxy aeginetic acid 5-*O*-*β*-D-quinovoside (**68**)	isolated	roots	fresh	NMR, MS	[61]
rehmamegastigmane (**69**)	isolated	roots	fresh	NMR, MS	[35]
*neo*-rehmannioside (**70**)	isolated	roots	dried	NMR, MS	[21]
*sec*-hydroxyaeginetic acid (**71**)	isolated	root tubers	-	NMR, MS	[27]
	isolated	roots	fresh	NMR, MS	[62]
	isolated	roots	steamed	NMR, MS	[30]
frehmaglutin K (**72**)	isolated	roots	fresh	NMR, MS	[18]
frehmaglutin A (**73**)	isolated	roots	fresh	NMR, MS	[18]
	isolated	roots	air-dried	NMR, MS	[31]
rehmaglutin A_1_ (**74**)	isolated	roots	fresh	NMR, MS	[61]
frehmaglutoside H (**75**)	isolated	roots	dried	NMR, MS	[32]
frehmaglutoside I (**76**)	isolated	roots	dried	NMR, MS	[33]
*sec*-hydroxyaeginetic acid-2-*O*-*β*-D-glucopyranoside (**77**)	isolated	roots	fresh	NMR	[61]
rehmannia B (**78**)	isolated	root tubers	-	NMR, MS	[27]
*sec*-hydroxyaeginetic acid lactate (**79**)	isolated	roots	fresh	NMR, MS	[61]
jiocarotenoside A_1_ (**80**)	isolated	roots	dried	NMR, MS	[14]
	isolated	roots	dried	NMR, MS, X-ray	[34]
jiocarotenoside A_2_ (**81**)	isolated	roots	dried	NMR, MS	[34]
14-hydroxyaeginetic acid (**82**)	isolated	roots	fresh	NMR	[61]
frehmaglutin C (**83**)	isolated	roots	fresh	NMR, MS	[18]
	isolated	roots	air-dried	NMR, MS	[31]
dihydroxy-*β*-ionone (**84**)	isolated	rhizomes	dried	NMR, MS	[13]
	isolated	roots	fresh	NMR, MS	[62]
	isolated	roots	steamed	NMR, MS	[30]
henryionoside B (**85**)	isolated	roots	fresh	NMR	[61]
	isolated	roots	fresh	NMR, MS	[62]
rehmaionoside A (**86**)	isolated	rhizomes	dried	NMR, MS	[13]
	isolated	root tubers	-	NMR, MS	[27]
	isolated	roots	dried	NMR, MS	[34]
	isolated	roots	dried	NMR, MS	[38]
	isolated	roots	dried	NMR, MS	[37]
rehmaionoside B (**87**)	isolated	roots	dried	NMR, MS	[38]
	isolated	roots	dried	NMR, MS	[37]
rehmaionoside C (**88**)	isolated	root tubers	-	NMR, MS	[27]
	isolated	roots	dried	NMR, MS	[32]
	isolated	roots	dried	NMR, MS	[34]
	isolated	roots	dried	NMR, MS	[38]
	isolated	roots	dried	NMR, MS	[37]
frehmaglutin H (**89**)	isolated	roots	fresh	NMR, MS	[18]
trihydroxy-*β*-ionone (**90**)	isolated	roots	dried	NMR, MS	[62]
	isolated	roots	steamed	NMR, MS	[30]
melasmoside (**91**)	isolated	roots	fresh	NMR	[17]
oxyrehmaionoside B (**92**)	isolated	roots	dried	NMR	[21]
	isolated	roots	fresh	NMR, MS	[36]
rehmannia C (**93**)	isolated	tuberous root	-	NMR, MS	[27]
frehmaglutin I (**94**)	isolated	roots	fresh	NMR, MS	[18]
frehmaglutin J (**95**)	isolated	roots	fresh	NMR, MS	[18]
abscisic acid (**96**)	isolated	roots	fresh	NMR	[61]
(6*S*,9*R*)-roseoside (**97**)	isolated	roots	fresh	NMR	[17]
3-hydroxy-*β*-ionone (**98**)	isolated	roots	fresh	NMR, MS	[18]
aeginetic dioxolane acid (**99**)	isolated	roots	fresh	NMR, MS	[61]
rehmaionoside C1 (**100**)	isolated	roots	fresh	NMR, MS	[61]
frehmaglutin D (**101**)	isolated	roots	air-dried	NMR, MS	[31]
	isolated	roots	fresh	NMR, MS	[62]
frehmaglutin G (**102**)	isolated	roots	fresh	NMR, MS	[62]
**Iridoid + ionone**					
frehmaglutoside G (**103**)	isolated	roots	dried	NMR, MS	[32]
rehmaglutoside F (**104**)	isolated	roots	air-dried	NMR, MS	[22]
aeginetoyl ajugol 5″-*O*-*β*-D-quinovoside (**105**)	isolated	roots	dried	NMR, MS	[47]
rehmannioside F (**106**)	isolated	roots	dried	NMR, MS	[14]
rehmaglutoside I (**107**)	isolated	roots	air-dried	NMR, MS	[22]
rehmaglutoside H (**108**)	isolated	roots	air-dried	NMR, MS	[22]
rehmaglutoside G (**109**)	isolated	roots	air-dried	NMR, MS	[22]
6-*O*-*sec*-hydroxyaeginetoyl ajugol (**110**)	isolated	roots	dried	NMR, MS	[34]
	isolated	roots	dried	NMR	[21]
rehmannioside E (**111**)	isolated	roots	dried	NMR, MS	[14]
**Phenylethanoid glycosides**					
2-phenylethyl-*β*-D-glucoside (**112**)	isolated	roots	air-dried	NMR	[39]
jionoside C (**113**)	isolated	roots	steamed	NMR, MS	[44]
salidroside (**114**)	isolated	roots	dried	NMR, MS	[40]
2-phenylethyl *O*-*β*-D-xylopyranosyl-(1→6)-*β*-D-glucopyranoside (**115**)	isolated	roots	dried	NMR, MS	[40]
cistanoside G (**116**)	isolated	roots	air-dried	NMR	[39]
darendoside A (**117**)	isolated	roots	dried	NMR, MS	[10]
2-phenylethyl *O*-*β*-D-xylopyranosyl-(1→2)-*β*-D-glucopyranoside (**118**)	isolated	roots	dried	NMR, MS	[40]
forsythiaside (=plantamajoside, **119**)	isolated	leaves	-	NMR, MS	[43]
3,4-dihydroxy-*β*-phenethyl-*O*-*β*-D-glucopyranosyl-(1→3)-4-*O*-caffeoyl-*β*-*D*-glucopyranoside (=purpureaside B, **120**)	isolated	leaves	-	NMR, MS	[43]
3,4-dihydroxy-β-phenethyl-*O*-*β*-D-glucopyranosyl-(1→3)-*O*-*α*-L-rhamnopyranosyl-(1→6)-4-*O*-caffeoyl-*β*-D-glucopyranoside (**121**)	isolated	leaves	-	NMR, MS	[43]
verbasoside (**122**)	isolated	roots	dried	NMR, MS	[23]
isoacteoside (**123**)	isolated	roots	dried	NMR, MS	[23]
	reference matching	roots	fresh/dried/steamed	HPLC	[59]
acteoside (=verbascoside, **124**)	isolated	leaves	air-dried	NMR, MS	[8]
	isolated	roots	dried	NMR, MS	[23]
	reference matching	roots	fresh/dried/steamed	HPLC	[59]
	reference matching	roots	steamed	HPLC-MS^n^	[63]
	isolated	leaves	fresh	NMR, MS	[64]
	isolated	roots	-	NMR, MS	[42]
echinacoside (**125**)	reference matching	leaves and roots	air-dried	LC-MS	[8]
3,4-dihydroxy-*β*-phenethyl-*O*-*α*-L-rhamnopyranosyl-(1→3)-*O*-*β*-D-galatopyranosyl-(1→6)-4-*O*-caffeoyl-*β*-D-glucopyranoside (=purpureaside C, **126**)	isolated	roots	-	NMR, MS	[42]
jionoside E (**127**)	isolated	roots	steamed	NMR, MS	[44]
leucosceptoside A (**128**)	isolated	roots	dried	NMR, MS	[23]
	isolated	roots	dried	NMR, MS	[44]
jionoside A_1_ (**129**)	isolated	roots	dried	NMR, MS	[23]
	isolated	roots	dried	NMR, MS	[44]
	reference matching	roots	steamed	HPLC-MS^n^	[63]
jionoside A_2_ (**130**)	isolated	roots	steamed	NMR, MS	[44]
2′-acetylacteoside (**131**)	isolated	roots	steamed	NMR, MS	[44]
darendoside B (**132**)	isolated	roots	fresh	NMR	[35]
isomartynoside (**133**)	isolated	roots	dried	NMR, MS	[23]
jionoside D (**134**)	isolated	roots	steamed	NMR, MS	[44]
martynoside (**135**)	isolated	leaves	air-dried	HPLC, NMR, MS	[8]
	reference matching	roots	steamed	HPLC-MS^n^	[63]
jionoside B_1_ (**136**)	isolated	roots	dried	NMR, MS	[23]
	isolated	roots	dried	NMR, MS	[44]
*cis*-martynoside (**137**)	isolated	root tubers	-	NMR, MS	[27]
jionoside B_2_ (**138**)	isolated	roots	steamed	NMR, MS	[44]
cistanoside A (**139**)	isolated	roots	steamed	NMR, MS	[65]
brachynoside (**140**)	isolated	roots	dried	NMR, MS	[23]
**Phenylpropanoids and their glycosides**					
cinnamic acid (**141**)	reference matching	roots	dried	LC-MS^2^	[48]
cistanoside F (**142**)	isolated	roots	steamed	NMR, MS	[44]
	isolated	roots	steamed	NMR, MS	[65]
coniferin (**143**)	isolated	roots	dried	NMR	[41]
dihydroferulic acid (**144**)	isolated	roots	fresh	NMR	[35]
nepetoidin F (**145**)	isolated	roots	fresh	NMR, MS	[49]
jiofuran acid A (**146**)	isolated	roots	fresh	NMR, MS	[66]
**Flavonoids**					
apigenin (=versulin, **147**)	isolated	leaves	air-dried	HPLC, NMR, MS	[8]
luteolin (**148**)	isolated	leaves	dried	NMR, MS	[67]
chrysoeriol (**149**)	isolated	leaves	air-dried	HPLC, NMR, MS	[8]
**Lignans**					
lariciresinol (**150**)	isolated	roots	fresh	NMR	[35]
lariciresinol 4′-*O*-*β*-D-glucopyranoside (**151**)	isolated	roots	fresh	NMR	[35]
isolariciresinol (**152**)	isolated	roots	fresh	NMR, MS	[18]
isolariciresinol-9′-*O*-*β*-D-glucopyranoside (**153**)	isolated	roots	fresh	NMR, MS	[66]
pinoresinol (**154**)	isolated	roots	fresh	NMR, MS	[18]
balanophonin B (**155**)	isolated	roots	fresh	NMR, MS	[66]
(+)-paulownin (**156**)	isolated	roots	fresh	NMR, MS	[66]
8′-hydroxyl episesaminone (**157**)	isolated	roots	fresh	NMR, MS	[66]
*trans*-liovil (**158**)	isolated	roots	dried	NMR, MS	[10]
(–)-(7*R*,8*S*,7′*R*,8′*S*)-4,9,4′,9′-tetrahydroxy-3,3′-dimethoxy-7,7′-epoxylignan-9-*O*-*β*-D-xylopyranoside (**159**)	isolated	roots	fresh	NMR, MS	[66]
threo-guaiacylglycerol-*β*-ferulic acid ether (**160**)	isolated	roots	fresh	NMR, MS	[18]
rhemaneolignan F (**161**)	isolated	roots	fresh	NMR, MS	[18]
rhemaneolignan E (**162**)	isolated	roots	fresh	NMR, MS	[18]
rhemaneolignan A (**163**)	isolated	roots	dried	NMR, MS	[33]
rhemaneolignan B (**164**)	isolated	roots	dried	NMR, MS	[33]
hierochin D (**165**)	isolated	roots	fresh	NMR	[35]
invonoid B (**166**)	isolated	roots	fresh	NMR, MS	[18]
yemuoside YM1 (**167**)	isolated	roots	fresh	NMR	[35]
(1,1′-biphenyl)-3,3′-dipropanoic acid, 6,6′-dihydroxy-5,5′-dimethoxy-3,3′-dimethyl ester (**168**)	isolated	roots	fresh	NMR, MS	[66]
rhemaneolignan C (**169**)	isolated	roots	dried	NMR, MS	[33]
(7*R*,8*S*)-4,9-dihydroxy-3,3′-dimethoxy-7,8-dihydrobenzofuran-1′-propanalneolignan (=hedyotisol A, **170**)	isolated	roots	dried	NMR, MS	[10]
**Terpenoids**					
ascaridole epoxide (**171**)	in-house library matching	fibrous roots	air-dried	GC-MS	[68]
3-*oxo*-2,6,6-trimethylcyclohex-1-ene-1-carboxylic acid (**172**)	isolated	roots	fresh	NMR	[61]
rehmapicrogenin (**173**)	isolated	roots	fresh	NMR, MS	[61]
	isolated	roots	steamed	NMR, MS	[30]
rehmapicroside (**174**)	isolated	roots	dried	NMR, MS	[38]
	isolated	roots	dried	NMR, MS	[37]
3-methoxy-2,6,6-trimethylcyclohex-1-enecarboxylic acid (**175**)	isolated	roots	fresh	NMR, MS	[18]
frehmaglutin E (**176**)	isolated	roots	air-dried	NMR, MS	[31]
(4*S*)-ethoxy rehmapicrogenin (**177**)	isolated	roots	fresh	NMR, MS	[18]
rehmapicrogenin monomethyl ester (**178**)	isolated	roots	fresh	NMR	[61]
cedrol (**179**)	isolated	roots	air-dried	NMR	[39]
aromadendrene (**180**)	in-house library matching	fibrous roots	air-dried	GC-MS	[68]
ent-oplopanone (**181**)	isolated	roots	air-dried	NMR	[39]
1,8-dihydroxy-2,2,8-trimethyl-6-oxabicyclo [3.2.1]octane-7-carboxylic acid (**182**)	isolated	roots	dried	NMR, MS	[69]
dihydrophaseic acid (**183**)	isolated	roots	fresh	NMR	[61]
dihydrophaseic acid 4-*O*-*β*-D-glucopyranoside (**184**)	isolated	roots	air-dried	NMR	[31]
remophilanetriol E (**185**)	isolated	roots	fresh	NMR, MS	[19]
remophilanetriol G (**186**)	isolated	roots	fresh	NMR, MS	[19]
remophilanetriol F (**187**)	isolated	roots	fresh	NMR, MS	[19]
remophilanetriol A (**188**)	isolated	roots	fresh	NMR, MS	[45]
remophilanetriol (**189**)	isolated	roots	dried	NMR, MS	[60]
remophilanetriol B (**190**)	isolated	roots	fresh	NMR, MS	[45]
remophilanetriol H (**191**)	isolated	roots	fresh	NMR, MS	[19]
remophilanetriol I (**192**)	isolated	roots	fresh	NMR, MS	[19]
diincarvilone C (**193**)	isolated	roots	fresh	NMR, MS	[45]
diincarvilone D (**194**)	isolated	roots	fresh	NMR, MS	[45]
columbin (**195**)	in-house library matching	fibrous roots	air-dried	GC-MS	[68]
4-*epi*-isopimaric acid (**196**)	isolated	roots	air-dried	NMR	[39]
pomonic acid (**197**)	isolated	roots	fresh	NMR, MS	[18]
glutinolic acid (**198**)	isolated	roots	dried	NMR, MS	[47]
oleanolic acid (**199**)	isolated	roots	fresh	NMR, MS	[18]
ursolic acid (**200**)	isolated	roots	fresh	NMR, MS	[18]
micromeric acid (**201**)	isolated	roots	fresh	NMR, MS	[18]
pomolic acid (**202**)	isolated	roots	fresh	NMR, MS	[18]
euscaphic acid (**203**)	isolated	roots	fresh	NMR, MS	[18]
2*β*-hydroxypomolic acid (**204**)	isolated	leaves	dried	NMR, MS	[70]
asiatic acid (**205**)	isolated	leaves	dried	NMR, MS	[70]
glutinosalactone B (**206**)	isolated	leaves	air-dried	NMR, MS	[46]
glutinosalactone A (**207**)	isolated	leaves	air-dried	NMR, MS	[46]
glutinosalactone C (**208**)	isolated	leaves	air-dried	NMR, MS	[46]
*β*-sitosterol (**209**)	isolated	roots	steamed	NMR, MS	[71]
daucosterol (**210**)	isolated	roots	fresh	NMR	[18]
*β*-sitosterol palmitate (**211**)	isolated	roots	fresh	NMR, MS	[18]
allopregnane-3,7,11,20-tetra-one (**212**)	in-house library matching	fibrous roots	air-dried	GC-MS	[68]
**Alkaloids**					
pterolactam (**213**)	isolated	roots	fresh	NMR	[49]
rehmanalkaloid C (**214**)	isolated	roots	dried	NMR, MS	[10]
rehmanalkaloid B (**215**)	isolated	roots	dried	NMR, MS	[10]
rehmanalkaloid A (**216**)	isolated	roots	dried	NMR, MS	[10]
glutinosine A (**217**)	isolated	leaves	air-dried	NMR, MS	[29]
glutinosine B (**218**)	isolated	leaves	air-dried	NMR, MS	[29]
glutinosine C (**219**)	isolated	leaves	air-dried	NMR, MS	[29]
**Saccharides**					
ethyl *β*-D-glucopyranoside (**220**)	isolated	leaves	fresh	NMR	[43]
sucrose (**221**)	reference matching	roots	air-dried	HPLC	[50]
raffinose (**222**)	reference matching	roots	air-dried	HPLC	[50]
*n*-hexyl *O*-*β*-D-xylopyranosyl-(1→6)-*β*-D-glucopyranoside (**223**)	isolated	roots	air-dried	NMR, MS	[40]
phenyl-6-*O*-*β*-D-xylopyranosyl-*O*-*β*-D-glucopyranoside (**224**)	isolated	roots	dried	NMR, MS	[10]
2-methoxy-4-methylphenyl *O*-*β*-D-apiofuranosyl-(1→6)-*β*-D-glucopyranoside (**225**)	isolated	roots	air-dried	NMR, MS	[40]
stachyose (**226**)	reference matching	roots	air-dried	HPLC	[50]
	isolated	roots	air-dried	NMR, MS	[51]
rehmaglupentasaccharide B (**227**)	isolated	roots	air-dried	NMR, MS	[51]
rehmaglupentasaccharide D (**228**)	isolated	roots	air-dried	NMR, MS	[51]
rehmaglupentasaccharide E (**229**)	isolated	roots	air-dried	NMR, MS	[51]
rehmaglupentasaccharide F (**230**)	isolated	roots	air-dried	NMR, MS	[51]
rehmaglupentasaccharide G (**231**)	isolated	roots	air-dried	NMR, MS	[51]
verbascose (**232**)	isolated	roots	air-dried	NMR	[51]
rehmaglupentasaccharide A (**233**)	isolated	roots	air-dried	NMR, MS	[51]
rehmaglupentasaccharide C (**234**)	isolated	roots	air-dried	NMR, MS	[51]
5-hydroxymethyl-2-furaldehyde (=2-formyl-5-hydroxymethylfuran, **235**)	isolated	roots	steamed	NMR	[13]
	reference matching	roots	air-dried	HPLC	[59]
5-hydroxymethylfuroic acid (**236**)	isolation	roots	steamed	NMR	[24]
rehmanone C (**237**)	isolated	roots	air-dried	NMR, MS	[72]
rehmanone B (**238**)	isolated	roots	air-dried	NMR, MS	[72]
serratifolide F (**239**)	isolated	roots	fresh	NMR, MS	[17]
5-(*α*-D-glucopyranosyloxymethyl)-2-furancarboxaldehyde (**240**)	isolated	roots	steamed	NMR	[73]
5-(α-D-glucopyranosyl-(1→6)-*α*-D-glucopyranosyloxymethyl)-2-furancarboxaldehyde (**241**)	isolated	roots	steamed	NMR, MS	[73]
rehmanniaeketone C (**242**)	isolated	roots	steamed	NMR, MS	[74]
rehmanniaeketone A (**243**)	isolated	roots	steamed	NMR, MS	[74]
rehmanniaeketone B (**244**)	isolated	roots	steamed	NMR, MS	[74]
rehmanone A (**245**)	isolated	roots	air-dried	NMR, MS	[72]
1,1-dimethoxycyclohexane (**246**)	in-house library matching	fibrous roots	air-dried	GC-MS	[68]
2,4-dimethoxy-2-methyl-6H-pyran-3-one (**247**)	isolated	roots	air-dried	NMR, MS	[13]
*p*-hydroxyphenylethyl alcohol (=*p*-tyrosol, **248**)	isolated	roots	air-dried	NMR	[35]
	isolated	roots	air-dried	NMR	[39]
ferulic acid (**249**)	in-house library matching	fibrous roots	air-dried	GC-MS	[68]
vanillin (**250**)	in-house library matching	fibrous roots	air-dried	GC-MS	[68]
1,1′-(1,3-phenylene)diethanone (**251**)	in-house library matching	fibrous roots	air-dried	GC-MS	[68]
2,5-dihydroxyacetophenone (**252**)	isolated	roots	steamed	NMR, UV	[71]
4-hydroxy-3-methylacetophenone (**253**)	in-house library matching	fibrous roots	air-dried	GC-MS	[68]
**Miscellaneous constituents**					
benzoic acid (**254**)	in-house library matching	fibrous roots	air-dried	GC-MS	[68]
4-hydroxybenzoic acid (**255**)	in-house library matching	fibrous roots	air-dried	GC-MS	[68]
protocatechuic acid (**256**)	in-house library matching	fibrous roots	air-dried	GC-MS	[68]
vanillic acid (**257**)	isolated	roots	fresh	NMR	[35]
gallic acid (**258**)	in-house library matching	fibrous roots	air-dried	GC-MS	[68]
phthalic acid (**259**)	in-house library matching	fibrous roots	air-dried	GC-MS	[68]
salicylic acid (**260**)	in-house library matching	fibrous roots	air-dried	GC-MS	[68]
4-hydroxy-3-methoxy-benzenemethanediol (**261**)	isolated	roots	fresh	NMR, MS	[18]
*threo*-1-(4-hydroxy-3- methoxyphenyl)-1,2,3-propanetriol (**262**)	isolated	roots	fresh	NMR	[35]
2,3,5,4′-tetrahydroxystilbene-2-*O*-*β*-D-glucoside (THSG) (**263**)	isolated	roots	-	NMR	[75]
3,8-dihydroxy-2-(2-hydroxyethyl)chromone (**264**)	isolated	roots	steamed	NMR, MS	[74]
3,8-dihydroxy-2-[(2-*O*-*α*-D-galactopyranosyloxy)ethyl]chromone (**265**)	isolated	roots	steamed	NMR, MS	[74]
2,3-dihydrobenzofuran (**266**)	in-house library matching	fibrous roots	air-dried	GC-MS	[68]
fluorene (**267**)	in-house library matching	fibrous roots	air-dried	GC-MS	[68]
biliverdic acid (**268**)	isolated	roots	fresh	NMR	[49]
adenosine (**269**)	isolated	roots	air-dried	NMR	[39]
nicotinamide (**270**)	isolated	roots	fresh	NMR	[49]
nicotinic acid (**271**)	isolated	roots	fresh	NMR	[49]
uracil (**272**)	isolation	roots	steamed	NMR	[24]
uridine (**273**)	isolated	roots	air-dried	NMR	[39]
2′-*O*-methyluridine (**274**)	isolated	roots	fresh	NMR	[49]
1-*O*-ethyl-*β*-D-glucopyranosyl-(6→3′)-uridine (**275**)	isolation	roots	air-dried	NMR, MS	[69]
glycine (**276**)	reference matching	leaves	fresh	HPLC-UV	[76]
alanine (**277**)	reference matching	leaves	fresh	HPLC-UV	[76]
serine (**278**)	reference matching	leaves	fresh	HPLC-UV	[76]
cysteine (**279**)	reference matching	leaves	fresh	HPLC-UV	[76]
valine (**280**)	reference matching	leaves	fresh	HPLC-UV	[76]
threonine (**281**)	reference matching	leaves	fresh	HPLC-UV	[76]
leucine (**282**)	reference matching	leaves	fresh	HPLC-UV	[76]
asparagine (**283**)	reference matching	leaves	fresh	HPLC-UV	[76]
aspartatic acid (**284**)	reference matching	leaves	fresh	HPLC-UV	[76]
isoleucine (**285**)	reference matching	leaves	fresh	HPLC-UV	[76]
methionine (**286**)	reference matching	leaves	fresh	HPLC-UV	[76]
lysine (**287**)	reference matching	leaves	fresh	HPLC-UV	[76]
arginine (**288**)	reference matching	leaves	fresh	HPLC-UV	[76]
glutamine (**289**)	reference matching	leaves	fresh	HPLC-UV	[76]
glutamic acid (**290**)	reference matching	leaves	fresh	HPLC-UV	[76]
proline (**291**)	reference matching	leaves	fresh	HPLC-UV	[76]
5-oxoproline Na salt (**292**)	isolation	roots	steamed	NMR	[24]
tryptophan (**293**)	reference matching	leaves	fresh	HPLC-UV	[76]
histidine (**294**)	reference matching	leaves	fresh	HPLC-UV	[76]
phenylalanine (**295**)	reference matching	leaves	fresh	HPLC-UV	[76]
tyrosine (**296**)	reference matching	leaves	fresh	HPLC-UV	[76]
cystine (**297**)	reference matching	leaves	fresh	HPLC-UV	[76]
santolina epoxide (**298**)	in-house library matching	fibrous roots	air-dried	GC-MS	[68]
succinic acid (=butanedioic acid, **299**)	in-house library matching	fibrous roots	air-dried	GC-MS	[68]
L-hydroxybutanedioic acid (**300**)	in-house library matching	fibrous roots	air-dried	GC-MS	[68]
pentanoic acid (**301**)	in-house library matching	fibrous roots	air-dried	GC-MS	[68]
(5*R*)-(2*E*)-5-hydroxy-6-keto-2-methyl-2-heptenoic acid (**302**)	isolated	roots	fresh	NMR, MS	[18]
2′-hydroxyethyl (2*E*,4*E*)-2-methyl-6-oxohepta-2,4-dienoate (**303**)	isolated	roots	air-dried	NMR, MS	[69]
rehmagluamide (**304**)	isolated	roots	fresh	NMR, MS	[49]
heptyl ester butanoic acid (**305**)	in-house library matching	fibrous roots	air-dried	GC-MS	[68]
(*E*)-10-hydroxydec-2-enoic acid methyl ester 10-*O*-*α*-L-rhamnoside (**306**)	isolated	roots	air-dried	NMR, MS	[39]
dodecanoic acid (**307**)	in-house library matching	fibrous roots	air-dried	GC-MS	[68]
tetradecanoic acid (**308**)	in-house library matching	fibrous roots	air-dried	GC-MS	[68]
methyl ester pentadecanoic acid (**309**)	in-house library matching	fibrous roots	air-dried	GC-MS	[68]
methyl ester 11-hexadecenoic acid (**310**)	in-house library matching	fibrous roots	air-dried	GC-MS	[68]
(*E*)-phytol (**311**)	isolated	roots	air-dried	NMR	[39]
	isolated	roots	air-dried	NMR	[77]
linoleic acid ethyl ester (**312**)	in-house library matching	fibrous roots	air-dried	GC-MS	[68]
oleic acid (**313**)	in-house library matching	fibrous roots	air-dried	GC-MS	[68]
tianshic acid (**314**)	isolated	roots	air-dried	NMR	[69]
eicosane (**315**)	in-house library matching	fibrous roots	air-dried	GC-MS	[68]
jio-cerebroside (**316**)	isolated	roots	air-dried	NMR, MS	[25]

According to the Pharmacopoeia of the People’s Republic of China, rehmannioside D (**6**) and catalpol (**43**) are the key ingredients used to assess the quality of *R. glutinosa* and they decrease during steaming process [78]. Interestingly, derivatization at C-6 position of ajugol (**13**) has produced various compounds (**17**–**26**) in nature. The derivatives can be caffeoyl, cinnamoyl, coumaroyl, feruloyl, or vanilloyl moieties. Compounds **17**–**21** and **39**–**41** were identified as iridoid glycosides with phenylpropanoids. In particular, the structures of **40** and **41**, the C-8″ enantiomers, were similar to 6-*O*-(*E*)-caffeoyl ajugol (**17**), except for the additional phenylpropanoid group at C-4″ of feruloyl moiety [23]. Compounds **42**–**46** own an additional epoxide bridge between C-7 and C-8, instead of a double bond.

Rehmaglutoside J (**47**) and K (**48**) are isomeric bisiridoid bisglucosides, which are composed of ajugol (**13**) and an analog of ajugol through the connection of C-6′-O-C-3″ [22]. Compounds **49**–**55** are tricyclic iridoid structures with a 1,10-oxygen bridge [13,24,25,26], and one pair of additional double bonds was formed between C-3 and C-4 in rehmannia A (**55**), compared to rehmaglutin A (**52**) [27]. The structures of the iridoids and their glycosides are shown in Figure 1.

### 2.2. Ionones and Their Glycosides

The term “ionone” originated from “iona” (Greek for violet scent) and “ketone” (structure), and ionones are found in natural products as secondary metabolites [79]. In particular, ionone and their glycosides from *R. glutinosa* are formed by the breakdown of carotenoids and considered as a megastigmane-type C_13_ norisopernoid, which is composed of 13 carbon atoms arranged in a trimethylcyclohexane ring with a four-carbon side chain [80]. Compounds **63**–**102** belong to the ionone class, and the ionones in *R. glutinosa* are usually referred to “frehmaglutins”, “frehmaglutinoisdes”, “rehmaglutins”, or “rehmaionosides”.

The structures of ionones and their glycosides are shown in Figure 2.

Compounds **103**–**111** belong to iridoid–ionone conjugates with glycosides, where an iridoid (ajugol, **13**) is esterified with a side chain derived from aeginetic acid (**63**) or its derivatives. The rehmanniosides (**106** and **111**) and rehmaglutosides (**104** and **107**–**109**) were isolated from *R. glutinosa* for the first time and considered as specific chemical markers for identifying this plant in herbal mixtures. Compounds **103**–**111** contributed to the broad pharmacological properties, including anti-inflammatory, hepatoprotective, and immune-stimulating effects. The structures of iridoid–ionone conjugates are shown in Figure 3.

### 2.3. Phenylethanoid Glycosides

Phenylethanoid glycosides are the secondary metabolites usually found in plants of the order Lamiales [81], and they contain various substituents such as caffeic acid, ferulic acid, and saccharides [82]. Acteoside (verbascoside, **124**) is one of the representative phenylethanoid glycosides distributed in over 150 species [81]. In 2025, Ma et al. elucidated the structure of a uridine diphosphate glycosyltransferase from *R. glutinosa*, which plays an important role in the biosynthesis of phenylethanoid glycosides by catalyzing the regioselective rhamnosylation of the 3′-OH [83]. Jionoside A_1_ (**129**), its isomer jionoside A_2_ (**130**), jionoside B_1_ (**136**), and its isomer jionoside B_2_ (**138**) share structural features, including a rhamnose at R_5_, an (*E*)- or (*Z*)-feruloyl moiety at R_6_, and galactose at R_7_ [44,65]. The structures of ethanoid glycosides from *R. glutinosa* are shown in Figure 4.

### 2.4. Phenylpropanoids and Their Glycosides

Phenylpropanoids are secondary metabolites derived from L-phenylalanine by L-phenylalanine ammonia-lyase (PAL), and the phenylpropanoid pathway remains a rich source of metabolites including flavonoids and lignin [84]. While phenylpropanoids (**141**–**146**, Figure 5) are structurally related to the phenylethanoids, they lack the ethyl bridge and typically possess the characteristic cinnamic acid backbone. Nepetoidin F (**145**) is a hydroxycinnamic acid derivative, a conjugated form of one phenylpropanoid and one phenylethanoid [49].

### 2.5. Flavonoids

Flavonoids are polyphenolic constituents abundantly found in fruits, vegetables, tea, and wine. Although *R. glutinosa* has been known for its iridoid and phenylethanoid glycosides, flavonoids show relatively limited structural diversity in this plant. Apigenin (**147**) is a trihydroxyflavone (4′,5,7-trihydroxyflavone) and has been known for its effects on diabetes, amnesia, depression, and cancer [85]. Luteolin (**148**) has been widely used as a yellow dye [86]. The flavonoids from *R. glutinosa* are shown in Figure 5.

### 2.6. Lignans

Lignans are related to lignin biosynthesis and can also contribute to plant defense against fungi, bacteria, and insects. Compounds **150**–**159** (Figure 6) are lignans formed by the dimerization of two phenylpropanoid units (C_6_-C_3_) between a *β*-*β*′ linkage, and compounds **160**–**169** are considered as a class of neolignans in which two phenylpropane units are connected through linkages other than the conventional C-8-C-8′ bond found in classic lignans [87]. Lariciresinol (**150**) and isolariciresinol (**152**) are tetrahydrofuran and tetrahydronaphthalene types, respectively, and thus constitutional isomers. Feng, W.’s research group has worked on *R. glutinosa* and found unique neolignans, which are called rhemaneolignans (**161**–**164** and **169**) [18,33]. The ^1^H and ^13^C NMR data of rhemaneolignan F (**161**) and E (**162**) are similar to those of asprenol B from *Ilex asprella,* an *α*,*β*-unsaturated keto group at C-1′ instead of a propyl alcohol [88]. Rhemaneolignans A (**163**) and B (**164**) are oxyneolignan types, and rhemaneolignan C (**169**) is *β*-glucopyranosylated daphneresinol [33]. Hedyotisol A (**170**) was isolated from *Hedyotis lawsoniae* for the first time in 1984, is biosynthetically constructed from a syringaresinol unit and two phenylpropanoid units, and is thus considered as a furanoid dilignan structure [89]. Recent research has used this compound to investigate the thermal deconstruction behavior of *β*-*O*-4 linkage in lignins [90].

### 2.7. Terpenoids

Although *R. glutinosa* is one of the classic “iridoid plants” and its terpenoid profile focuses on monoterpenoid iridoid and their glycosides, various classes of terpenoids are found from this plant. Most of the monoterpenoids (**172**–**178**) from *R. glutinosa* share the skeleton of *β*-cyclogeranic acid, which is a trimethylcyclohexene ring with a carboxylic acid moiety. Interestingly, compounds **173**–**178** are the “rehmapicro” family with the same monoterpene skeleton.

Compounds **179**–**194** are involved in sesquiterpenoids. Eremophilane-type sesquiterpenoids (**185**–**192**), characterized by the existence of a bicyclic backbone, are especially abundant in this plant [19,45]. Interestingly, dimeric eremophilane compounds (**193** and **194**), firstly found in *Incarvillea arguta* (Bignoniaceae), were also isolated from *R. glutinosa* [45,91]. Furthermore, two diterpenoids were identified in *R. glutinosa*: columbin (**195**) and 4-*epi*-isopimaric acid (**196**).

Among the triterpenoids (**197**–**212**) in *R. glutinosa*, glutinosalactones A–C (**206**–**208**) were found to be ursane-type triterpenes with the presence of an *δ*-lactone ring between C-20 and C-18 [46]. The structures of terpenoids from *R. glutinosa* are shown in Figure 7.

### 2.8. Alkaloids

Various types of alkaloids have been reported from *R. glutinosa*. Shi et al. reported three new pyridine-type alkaloids, glutinosines A−C (**217**–**219**), isolated from *R. glutinosa* [29]. The ^1^H and ^13^C NMR data of these constituents were similar to those of rehmachinin C isolated from *R. chingii*, and the structural differences were an additional *N*-leucine group for glutinosines A and B and an additional *N*-phenylalanine group for glutinosine C, respectively [92]. The structures of alkaloids from *R. glutinosa* are presented in Figure 8.

### 2.9. Saccharides

Saccharides are the most abundant components in *R. glutinosa,* and their content can be increased during processing. Monosaccharides and disaccharides (**220**–**225**) serve as basic energy-related metabolites in the plant and contribute to the sweetness of the processed *R. glutinosa* root [7]. Oligosaccharides (**226**–**234**) have been known to be important for the medicinal effects of *R. glutinosa*. The interesting characteristic of the oligosaccharides from *R. glutinosa* is that they have stachyose (**226**), a tetrasaccharide consisting of two *α*-D-galactose units, one *α*-D-glucose unit, and one *β*-D-fructose unit sequentially linked as Gal(*α*1→6)Gal(*α*1→6)Glc(*α*1↔2*β*)Fruf, as a structural unit [93]. Rehmaglupentasaccharides (A–G) (**227**–**231**, **233**, and **234**) are the specialized oligosaccharides named after the *Rehmannia* genus and show the structural diversity of sugars in this plant [51]. They (0.2%, *w*/*w*) exhibited a moderate proliferation effect on *Lactobacillus reuteri*. The structures of saccharides are shown in Figure 9.

### 2.10. Other Compounds

Furan derivatives (**235**–**245**, Figure 10) are primarily volatile components or decomposition products often found in prepared *R. glutinosa*, and thus, 5-hydroxymethyl-2-furaldehyde (**235**) is the standard quality marker for processed *Rehmannia* and its concentration increases during steaming [94]. Rehmanones A–C (**237**, **238**, **and 245**) were named after the *Rehmannia* genus and contribute to the unique chemical properties of this plant [72]. Among amino acids (**276**–**297**), a sulfur-containing amino acid (cystine, **297**) was formed by the oxidation of two cysteine molecules (**279**) by a disulfide bond. The disulfide linkages in cysteine give strength and permanent shape to proteins such as keratin and their associated proteins [95].

Fatty acids and esters (**301**–**316**) are lipid components that can contribute to the nutritional and restorative properties of plants. (*E*)-phytol (**311**) is released during chlorophyll degradation and contributes to vitamin E biosynthesis through its conversion to phytyl diphosphate [96]. Oleic acid (**313**) is an unsaturated fatty acid/ester and well known to be a “healthy fat”, which is essential for maintaining skin barrier function and cardiovascular health [97]. The structures of miscellaneous compounds from *R. glutinosa* are shown in Figure 11.

## 3. Pharmacological Activities

Except for the cytotoxic, antiproliferative, and antioxidant properties of the identified compounds from *R. glutinosa*, most of their biological activities have been studied using rodent in vitro and in vivo models. The pharmacological activities discussed in this review are summarized in Table 2.

### 3.1. Antidiabetic and Anti-Obesity Activities

2,3,5,4′-tetrahydroxystilbene-2-*O*-*β*-D-glucoside (**263**, 0, 40, and 200 μg/mL) exhibited non-competitive, reversible inhibition of α-glucosidase (IC_50_ = 0.186 ± 0.003 μmol/mL), showing stronger potency than the positive control acarbose (IC_50_ = 5.42 ± 0.09 μg/mL) [75]. Compound **263** increased the cell viability of INS-1 cells from 52.13% to 76.26% in the oxidative stress model. Furthermore, it reduced the content of malondialdehyde (MDA), the biomarker of oxidative stress, from 213.68% to 67.47% of the normal group at 80 μM and enhanced the total amount of superoxide dismutase (SOD) by 26.27%. Compound **263** also increased the total insulin content by 59.37% compared with the model group. These results suggested that **263** modulated *α*-glucosidase inhibition, oxidative damage to pancreatic *β*-cells, and insulin secretion, thereby contributing to the regulation of blood glucose levels.

Catalpol (**43**) alleviated hepatic insulin resistance in high-fat diet (HFD)/streptozotocin (STZ)-induced type 2 diabetic mice (200 mg/kg/d) and HepG2 cells (20–80 μM) [98]. Compound **43** reduced fasting blood glucose, improved glucose tolerance and insulin sensitivity, increased serum insulin and hepatic glycogen content, and suppressed hepatic gluconeogenesis both in vivo and in vitro. Compound **43** attenuated NOX4-mediated oxidative stress by downregulating NOX4 protein expression and enhancing AMPK and PI3K/AKT phosphorylation, thereby improving hepatic insulin resistance. Huang et al. also reported that catalpol (0.1 mg/kg, i.v.) significantly reduced the level of plasma glucose in STZ-induced diabetic rats and stimulated the uptake of radioactive glucose [99]. The glucose-lowering effect was dose-dependent between 0.01 and 0.1 mg/kg (a minimal reduction of 8.53 ± 2.85% and a maximal reduction of 24.33 ± 2.94%), with no further effect on raising the dose to 0.15 mg/kg.

*sec*-Hydroxyaeginetic acid (**71**), rehmaionoside A (**86**), and rehmaionoside C (**88**) exhibited moderate α-glucosidase inhibitory activity, with IC_50_ values of 5.0, 3.1, and 6.3 mM, respectively, compared with acarbose (IC_50_ = 3.0 mM) [27]. Acteoside (**124**), leucosceptoside A (**128**), jionoside A_1_ (**129**), jionoside B_1_ (**136**), and brachynoside (**140**) exhibited moderate α-glucosidase inhibitory activity, with IC_50_ values ranging from 261.4 to 408.7 μM, compared with a positive control, acarbose (IC_50_ = 204.2  ±  19.9 μM) [23].

**Table 2:** Summary of biological activities of the constituents from the roots of *R. glutinosa*.

Compound/ Extract	Plant Source	Evidence Level	Model	Dose/ Concentration	Comparator	Main Outcome	Proposed Mechanism	References
Iridoids and their glycosides								
aucubin (**1**)	*Plantago asiatica*; isolated compound	in vitro	hepatitis B-transfected HepG2 cells	30, 100, 300, and 1000 μM (±β-glucosidase), 9 d; cytotoxicity 300–10 000 μM	untreated control; 2′,3′-dideoxycytidine (25 μM)	aucubin alone, weak (3.0-fold ↓extracellular HBV DNA at 1000 μM, intracellular unchanged); with β-glucosidase (100–1000 μM) >80-fold ↓virion DNA and >25-fold ↓replicating intermediates; no cytotoxicity ≤ 1000 μM	β-glucosidase-dependent conversion to aglycone; suppression of HBV DNA replication	[100]
	commercially purchased	in vitro	TNF-α-treated 3T3-L1 adipocytes	1, 3, 10, and 30 μM, 6 h pretreatment	TNF-α-stimulated control; PD98059 (ERK inhibitor, 20 μM)	↓TNF-α-induced MCP-1, PAI-1, IL-6 secretion (IC_50_ = 8.20, 9.94, 6.85 μM); at 10 μM ↓MCP-1, PAI-1, IL-6 mRNA by 36.5%, 24.3%, 39.0%; ↓nuclear p65	↓ERK1/2 phosphorylation; prevention of IκBα phosphorylation/degradation; ↓NF-κB nuclear translocation	[91]
	commercially purchased	in vitro	LPS- treated HepG2 cells	0–240 μM (viability), 2 h pretreatment before LPS, 24 h	LPS control; silymarin	↓TNF-α, IL-6, NO and iNOS activity; ↓ROS, MDA; ↑SOD, GSH-Px in LPS-induced HepG2 cells, concentration-dependent (*p* < 0.001); no cytotoxicity up to 240 μM	↓STAT3 phosphorylation and NF-κB (p65) nuclear transfer; NRF2/HO-1 upregulation	[101]
	commercially purchased	in vivo	male C57BL/6 mice, LPS-induced acute hepatitis	25, 50, and 100 mg/kg, i.p., 3 d	LPS control; silymarin (100 mg/kg, i.p.)	↓serum TNF-α, IL-6 dose-dependently; ↓hepatic MDA; ↑SOD, GSH-Px; improved hepatic histopathology at 50 and 100 mg/kg (25 mg/kg weaker); ↓nuclear NF-κB p65 and p-STAT3; ↑nuclear NRF2, HO-1	STAT3/NF-κB inhibition; NRF2/HO-1 activation	[101]
	commercially purchased	in vitro	H_2_O_2_-treated primary mouse cortical neurons (C57BL/6)	50, 100, and 200 μg/mL, added at 0 and 6 h after H_2_O_2_, 12 h exposure	H_2_O_2_ control; no positive control	dose-dependent, ↑nuclear Nrf2, NQO-1, HO-1, and Bcl2 and ↓Bax; cleaved caspase-3 in H_2_O_2_-injured primary cortical neurons; 200 μg/mL reversed H_2_O_2_-induced ROS rise (*p* < 0.05)	Nrf2 nuclear translocation → HO-1/NQO-1 antioxidant response; ↓apoptosis	[102]
	commercially purchased	in vivo	male C57BL/6 mice, weight-drop traumatic brain injury	20 and 40 mg/kg, i.p., at 30 min, 12 h, 24, h and 48 h after TBI	TBI control; no positive control + Nrf2-shRNA lentivirus as blocker	↓brain water content and MMP-9; improved mNSS, rotarod, and Morris water maze performance; ↑SOD, GSH, GSH-Px, ↓MDA; ↓TUNEL^+^ cells, microglial recruitment, HMGB1/TLR4/MyD88 and nuclear NF-κB p65 (*p* < 0.05)	Nrf2 activation (HO-1, NQO-1); ↓HMGB1–TLR4–MyD88–NF-κB inflammatory signaling; effects abolished by Nrf2 knockdown	[102]
	commercially purchased	in vivo	male albino rats, nonylphenol-induced testicular damage	5 mg/kg, i.p., 56 d	nonylphenol (100 μg/kg, i.p.) control; no positive control	↑GSR, CAT, SOD, GPx, TPC; ↓ROS, TBARS; ↑FSH, LH, testosterone, 3β-HSD, 17β-HSD; ↑sperm count, motility, membrane integrity; ↓dead/abnormal sperm; restored testicular histomorphometry (*p* < 0.05)	antioxidant and anti-apoptotic action: ↑Bcl-2, ↓Bax, caspase-9, caspase-3; restored steroidogenesis	[103]
monomelittoside (**3**)	*R. glutinosa*; isolated compound	in vitro	DOX-treated rat H9c2 cardiomyocytes	50 μM, 24 h	DOX control; no positive control	↑viability of DOX-injured cardiomyocytes; * *p* < 0.05, ** *p* < 0.01 vs. DOX group	-	[10]
rehmaglutoside L (**5**)	*R. glutinosa*; isolated compound	in vitro	DOX-treated rat H9c2 cardiomyocytes	50 μM, 24 h	DOX control; no positive control	↑viability of DOX-injured cardiomyocytes; * *p* < 0.05, ** *p* < 0.01 vs. DOX group	-	[10]
rehmannioside D (**6**)	*R. glutinosa*; isolated compound	in vivo	male C57BL/6J mice, CCl_4_ liver fibrosis/Wistar rats, DMN liver fibrosis	10 and 40 mg/kg, i.g., 3 w (CCl_4_ mice) and 2 w (DMN rats)	CCl_4_/DMN control; sorafenib (7.2 mg/kg), polyene phosphatidylcholine (273.6 mg/kg)	↓serum ALT, AST; ↓Adgre1, Tnfα, Il1β, Ccl2; ↓Sirius red^+^ area, hepatic Hyp, α-SMA, Acta2, Col1α1; ↓TUNEL^+^ hepatocytes, acetyl-p53, Bax/Bcl2, cleaved caspase-3; ↑Sirt7 (*p* < 0.05, *p* < 0.01)	Sirt7 restoration → ↓p53 acetylation/p53 signaling → ↓hepatocyte apoptosis; ↓HSC activation	[104]
geniposide (**11**)	commercially purchased	in vitro; in silico	TNF-α-induced RA-FLS (human rheumatoid synoviocytes)	0, 25, 50, and 100 μM, 24 h; IC_50_ = 67.47 μM (24 h) and 31.76 μM (48 h) for RA-FLS proliferation; cytotoxic to HFLS at 200 μM	TNF-α control; no positive control	↓RA-FLS proliferation dose-dependently (IC_50_ = 67.47 μM/24 h, 31.76 μM/48 h); ↓IL-17, IL-8, TNF-α, MMP-3, MMP-9 concentration-dependently; ↓p-JAK1, p-STAT1	inhibition of JAK1/STAT1 phosphorylation (JAK-STAT signaling); docking to 12 hub targets (−7.51 to −11.6 kcal/mol)	[105]
	commercially purchased	in vivo	male C57BL/6 mice, collagen-induced arthritis	60 and 120 mg/kg, i.p., 14 d	CIA control; methotrexate (0.5 mg/kg)	↓hind-paw swelling and weight loss; ↓serum IL-17, TNF-α, IL-8, MMP-3, MMP-9; ↑IL-10; ↓cartilage destruction and inflammatory-cell infiltration in CIA mice	inhibition of JAK-STAT (JAK1/STAT1) signaling	[105]
	commercially purchased	in vivo	male Wistar rats, STZ/HFD diabetic wound	200, 400, and 500 mg/kg, p.o., 7 d	diabetic control; no positive control	↑lesion retraction 1.06–1.84× on d 7; ↓blood glucose (HbA1c unchanged); ↓inflammatory-cell infiltration 16.4% and 18.9%; ↑fibroblast proliferation 27.0%, 34.5%, 46.0%; ↓TNF-α, IL-1β, IL-6 (IC_50_ = 1.36, 1.02, 1.23 g/kg); ↑IL-10 1.14-, 1.57-, 1.86-fold	anti-inflammatory action via IL-10 upregulation suppressing pro-inflammatory cytokines (IKK/NF-κB suggested)	[106]
	commercially purchased	in vivo	male C57BL/6 mice, TAC- and isoprenaline-induced hypertrophy	25 or 50 mg/kg, p.o., 7 w (TAC model); 50 mg/kg, 14 d (isoprenaline model)	vehicle control; no positive control	↓HW/BW, HW/TL and cardiomyocyte cross-sectional area dose-dependently; ↑ejection fraction and FS; ↓LVIDd; ↓ANP, β-MHC, XBP-1s; ↑p-AMPKα; ↓p-mTOR, p-ERK, ER stress (*p* < 0.05)	GLP-1 receptor/AMPKα signaling, with downstream mTOR, ERK, and ER-stress inhibition	[107]
	-	in vivo	Male C57BL/6 mice, TAC-induced myocardial fibrosis	10, 20, and 40 mg/kg, p.o., 4 w	TAC control; no positive control	dose-dependent, ↑EF (33.47 ± 11.81% TAC → 58.74, 62.64, 66.61%) and FS (22.87 → 26.12, 37.66, 42.50%); ↓LV mass (182.67 → 165.55, 155.82, 141.91 mg) and LVPWD; ↓interstitial fibrosis, BNP, ANP, collagen I/III, α-SMA, MMP-9; ↓TGF-β1, p-Smad2 (*p* < 0.01)	suppression of TGF-β1/Smad2 signaling and MAPK (AKT/ERK) activation	[108]
ajugol (**13**)	*R. chingii*; isolated compound	in vitro	APAP-treated human HepG2 cells	10 μM, 48 h	APAP control; bicyclol (10 μM)	hepatoprotective activity; ↑cell survival to 69.9 ± 3.6% of normal vs. 58.5 ± 3.7% APAP control (*p* < 0.001); exceeded bicyclol	-	[109]
	*Sideritis lycia*; isolated compound	in vitro	ATCC bacterial and yeast strains (microdilution MIC)	wwo-fold dilutions 3.125–500 μg/mL; MIC 31.25–62.5 μg/mL	untreated control; ampicillin (MIC 0.98–3.9 μg/mL), oxiconazole (MIC 7.8 μg/mL)	MIC 31.25 μg/mL against *Bacillus subtilis*, *Staphylococcus aureus*, and *Escherichia coli*; MIC 62.5 μg/mL against *Pseudomonas aeruginosa*, *Candida albicans*, and *Cryptococcus neoformans*	-	[110]
ajugoside (14)	commercially purchased	in vivo	female C57BL/6J mice, ovariectomy-induced osteoporosis	100, 200, and 400 mg/kg, p.o., 4 w	OVX control; resveratrol (20 mg/kg/d)	dose-dependently ↑BV/TV, Tb.N, Tb.Th; ↓Tb.Sp; ↓bone marrow fat; ↑OCN+ cells; ↑serum SOD and GSH-Px; ↓MDA; ↓Slc5a1; ↑p-AMPK/AMPK, p-ULK1/ULK1, LC3B-II/I	Slc5a1 inhibition → AMPK-mediated mitophagy, ↓oxidative stress; direct ajugoside–Slc5a1 binding shown by pull-down	[111]
rehmaglutoside C (**26**)	*R. glutinosa*; isolated compound	in vitro	D-GalN-treated HL-7702 hepatocytes	10 μM, 1 h pretreatment	D-GalN control; bicyclol (10 μM)	↑cell survival to 71.0 ± 1.9% of normal (*p* < 0.01); 33.6% inhibition of D-GalN-induced injury, exceeding bicyclol; non-cytotoxic, IC_50_ > 10 μM	-	[22]
(8*S*)-7,8-dihydrogeniposide (**31**)	*R. glutinosa*; isolated compound	in vitro	DOX-treated rat H9c2 cardiomyocytes	50 μM, 24 h	DOX control; no positive control	↑viability of DOX-injured cardiomyocytes; *p* < 0.05, *p* < 0.01 vs. DOX group	-	[10]
11-methylforsythide (**32**)	*R. glutinosa*; isolated compound	in vitro	LPS-treated NRK-52E rat kidney tubular epithelial cells	10 μM; 0.1–100 μM dose–response; EC_50_ = 45.1 μM	LPS control; no positive control	↑viability of LPS-injured cells to 0.772 ± 0.051 at 10 μM (*p* < 0.001 vs. LPS), restoring normal control level; EC_50_ = 45.1 μM	-	[17]
mussaenoside (**33**)	*R. glutinosa*; isolated compound	in vitro	DOX-treated rat H9c2 cardiomyocytes	50 μM, 24 h	DOX control; no positive control	↑viability of DOX-injured cardiomyocytes; *p* < 0.05, *p* < 0.01 vs. DOX group	-	[10]
	*R. glutinosa*; isolated compound	in vitro	TGF-β1-treated BEAS-2B bronchial epithelial cells	10 μM, 24 h	TGF-β1 control; no positive control	↑viability of TGF-β1-injured cells: 1.061 ± 0.019 vs. 1.000 ± 0.012 model (*p* < 0.05)	-	[19]
8-epiloganin (**35**)	*R. glutinosa*; isolated compound	in vitro	TGF-β1-treated BEAS-2B bronchial epithelial cells	10 μM, 24 h	TGF-β1 control; no positive control	↑viability of TGF-β1-injured cells: 1.077 ± 0.004 vs. 1.000 ± 0.012 model (*p* < 0.01)	-	[19]
gardoside methyl ester (**37**)	*R. glutinosa*; isolated compound	in vitro	TGF-β1-treated BEAS-2B bronchial epithelial cells	10 μM, 24 h	TGF-β1 control; no positive control	↑viability of TGF-β1-injured cells: 1.093 ± 0.005 vs. 1.000 ± 0.012 model (*p* < 0.01)	-	[19]
catalpol (**43**)	commercially purchased	clinical	patients after surgical resection for locally advanced colon cancer (*n* = 115 per arm; 48-month follow-up)	10 mg/kg, i.p., twice daily, 12 w	no-chemotherapy group; bevacizumab (5 mg/kg, i.v., twice weekly)	proportion of patients with CA 19-9 > 27 ng/L fell from 88% to 77% (*p* = 0.0002) and CEA > 5 ng/L from 79% to 69% (*p* = 0.0002); serum MMP-2 92.27 ± 10.21 → 87.55 ± 5.39 and MMP-9 225.25 ± 6.23 → 223.15 ± 5.31 (both *p* < 0.0001); overall survival higher than the no-chemotherapy group (*p* < 0.0001) and comparable to bevacizumab (*p* = 0.3434)	downregulation of MMP-2/MMP-9	[112]
	*R. glutinosa*; isolated compound	in vitro	APAP-treated human HepG2 cells	10 μM, 48 h	APAP control; bicyclol (10 μM)	↑cell survival to 69.1 ± 0.7% of normal vs. 58.5 ± 3.7% APAP control (*p* < 0.001); exceeded bicyclol	-	[109]
	commercially purchased	in vitro	glucosamine- treated insulin-resistant HepG2 cells	20, 40, and 80 μM, 24 h (also 10 and 160 μM; 40 μM in inhibitor/siRNA experiments)	glucosamine control; no positive control + LY294002 as blocker	↑viability at 20–80 μM; ↓glucose production, PEPCK, G6pase, ROS, MDA, NOX4, p-GS (Ser641); ↑GSH, SOD, glycogen, p-AMPK, p-AKT 2.49-fold (1.71-fold with LY294002), p-FOXO1 2.29-fold, p-GSK3β 2.55-fold; NOX4 ↓2.91-fold control siRNA vs. 1.17-fold AMPK siRNA	AMPK/NOX4/PI3K/AKT; ↓ROS, FOXO1-driven gluconeogenesis; ↑GSK3β-dependent glycogen synthesis	[98]
	commercially purchased	in vivo	male C57BL/6J mice, HFD/STZ type 2 diabetes	100 and 200 mg/kg, p.o., 4 w	diabetic control; no positive control	↓fasting glucose, p-IRS-1 (Ser307), PEPCK, G6pase, p-GS (Ser641), MDA, NOX4, serum ALT, AST, hepatic steatosis; ↑insulin, OGTT/ITT response, p-AKT, p-AMPK, p-GSK3β, liver glycogen, GSH, SOD; body weight unchanged	hepatic AMPK/NOX4/PI3K/AKT–IRS-1; ↓oxidative stress and FOXO1 gluconeogenesis; ↑glycogen synthesis	[98]
	*R. glutinosa*; isolated compound	in vivo; in vitro	male Wistar rats, STZ-induced diabetes/isolated soleus muscle	0.01–0.15 mg/kg, i.v. (0.1 mg/kg effective); 10^−8^–10^−6^ M ex vivo	vehicle control; metformin (100 mg/kg)	↓plasma glucose 8.53 ± 2.85% (0.01 mg/kg) to maximal 24.33 ± 2.94% (0.1 mg/kg); ↓glucose rise at 5 and 20 min post-challenge; ↑soleus 2-DG uptake 26.55 ± 0.90 → 33.08 ± 1.62 pmol/mg protein/5 min; ↑hepatocyte glycogen synthesis	↑peripheral muscle glucose uptake and hepatic glycogen synthesis, insulin-independent; molecular target not identified	[99]
	commercially purchased	in vivo	male Wistar rats, isoproterenol-induced myocardial infarction	5 and 10 mg/kg, i.p., 10 d (pretreatment)	ISO control; no positive control	At 10 mg/kg ↑ + LVdp/dtmax (2730.14 ± 278.57→3346.38 ± 306.01 mmHg/s), ↑LVSP (122.71 ± 11.43→143.38 ± 10.39 mmHg), ↓LVEDP (17.34 ± 1.09→11.34 ± 1.15 mmHg); ↑plasma/myocardial apelin and APJ; ↓TUNEL-positive cells, caspase-3/9	↑apelin/APJ signaling; ↑Bcl-2/↓Bax → ↓caspase-3/9 apoptosis	[113]
	commercially purchased	in vivo	male Sprague–Dawley rats, pMCAO stroke	5 mg/kg, i.p., from 24 h after stroke, 7 d	MCAO control; no positive control + AG490 (5 mg/kg) as blocker	↓rCBF hemispheric difference (model 89.39 ± 1.55→52.8 ± 3.5, *p* < 0.05); ↑VWF/PCNA co-localized microvessels (*p* < 0.05); ↑pSTAT3 nuclear translocation, STAT3–VEGF promoter binding, VEGF mRNA/protein; improved limbuse asymmetry and beam walking	A JAK2/STAT3 → STAT3 binding to VEGF promoter → ↑VEGF → angiogenesis	[114]
	commercially purchased	in vivo; in vitro	male BALB/c mice, LPS-induced acute lung injury/LPS-stimulated MH-S alveolar macrophages	2.5, 5, and 10 mg/kg, i.p., single dose 1 h before LPS (10 mg/kg also 1 h after); 0.1, 0.3, and 0.5 mM in vitro, 1 h pretreatment	LPS control; dexamethasone (5 mg/kg)	↓lung W/D ratio, MPO activity, and BALF total cells, neutrophils, and macrophages (*p* < 0.01); ↓TNF-α, IL-6, IL-4, IL-1β, and ↑IL-10 in BALF and in LPS-stimulated MH-S alveolar macrophages (*p* < 0.05–0.01)	↓TLR4 → ↓IκB-α/NF-κB p65 and p38/JNK/ERK MAPK phosphorylation	[115]
	commercially purchased	in vivo; in vitro	4 Gy-irradiated human lymphocyte AHH-1 cells/male BALB/c mice, 8 Gy irradiation	25–100 μg/mL pretreatment before 4 Gy (AHH-1 cells); 25, 50, and 100 mg/kg, i.p., single dose 20 min before 8 Gy (mice)	irradiated control; no positive control	↑cell viability, ↓LDH leakage, and ↓apoptosis in 4 Gy-irradiated AHH-1 cells; ↓gastrointestinal Chiu damage by 15.6%, 33.3% and 44.4%; ↑plasma SOD (7.62 ± 0.67→10.06 ± 0.61 U/mg prot) and GSH, ↓MDA (6.81 ± 0.2→4.13 ± 0.13 nmol/mg), ↓8-OHdG; ↑WBC and platelets	ROS scavenging → ↑endogenous antioxidants, ↓lipid peroxidation, and ↓oxidative DNA damage	[116]
rehmaglutoside J (**47**)	*R. glutinosa*; isolated compound	in vitro	D-GalN-treated HL-7702 hepatocytes	10 μM, 1 h pretreatment	D-GalN control; bicyclol (10 μM)	↑cell survival to 67.3 ± 5.3% of normal (*p* < 0.01); 25.2% inhibition of D-GalN-induced injury; non-cytotoxic to five tumor cell lines, IC_50_ > 10 μM	-	[22]
rehmaglutoside K (**48**)	*R. glutinosa*; isolated compound	in vitro	D-GalN-treated HL-7702 hepatocytes	10 μM, 1 h pretreatment	D-GalN control; bicyclol (10 μM)	↑cell survival to 71.8 ± 2.9% of normal (*p* < 0.001); 35.5% inhibition of D-GalN-induced injury	-	[22]
rehmaglutin B (**53**)	*Russelia equisetiformis*; isolated compound	in vitro	LPS/IFN-γ-treated murine RAW264.7 macrophages	50 μM (final); IC_50_ = 52.8 ± 2.0 μM	LPS control; L-NMMA (50–100 μM)	↓NO production in LPS/IFN-γ-stimulated RAW264.7 cells, IC_50_ = 52.8 ± 2.0 μM, more potent than L-NMMA; no associated cytotoxicity (MTT)	-	[117]
rehmaglutin D (**54**)	*Adenosma caeruleum*; isolated compound	in vitro	LPS-treated BV2 microglial cells	Dose-dependent; IC_50_ = 53.1 ± 2.7 μM	LPS control; butein (IC_50_ = 4.5 ± 0.3 μM)	↓NO production in LPS-stimulated BV2 microglia, dose-dependent, IC_50_ = 53.1 ± 2.7 μM	-	[118]
jiofuran (**59**)	*Catalpa ovata*; isolated compound	in vitro	Nrf2-ARE dual-luciferase reporter 293T cells	25 μM, 24 h	DMSO control; EGCG (25 μM), gallic acid (25 μM)	significant Nrf2 agonistic activity in Nrf2 dual-luciferase reporter assay in 293T cells at 25 μM, approximately equal to positive controls; no numeric fold-change given	activation of Nrf2 transcriptional activity	[119]
**Ionones and their glycosides**								
frehmaglutin B (**64**)	*R. glutinosa*; isolated compound	in vitro	TGF-β1-treated BEAS-2B cells	10 μM	TGF-β1 control; no positive control	↑viability of TGF-β1-injured cells: 1.073 ± 0.007 vs. 1.000 ± 0.017 model (*p* < 0.05)	-	[61]
frehmaglutin B_1_ (**65**)	*R. glutinosa*; isolated compound	in vitro	TGF-β1-treated BEAS-2B cells	0.1–100 μM, 72 h; EC_50_ = 10.4 μM	TGF-β1 control; no positive control	↑viability 1.068 ± 0.016 vs. 1.000 ± 0.017 model (*p* < 0.05); EC_50_ = 10.4 μM (R = 0.939); ↓p-PI3K, p-AKT, p-p65, ROS, apoptosis, all reversed by 740Y-P	PI3K/AKT/NF-κB; ↓ROS and apoptosis	[61]
aeginetic acid 5-*O*-*β*-D-quinovoside (**66**)	*R. glutinosa*; isolated compound	in vitro	TGF-β1-treated BEAS-2B cells	10 μM	TGF-β1 control; no positive control	↑viability of TGF-β1-injured cells: 1.094 ± 0.015 vs. 1.000 ± 0.017 model (*p* < 0.05)	-	[61]
*sec*-hydroxyaeginetic acid (**71**)	*R. glutinosa*; isolated compound	in vitro	α-glucosidase (cell-free enzyme assay)	IC_50_ = 5.0 mM	untreated control; acarbose (IC_50_ = 3.0 mM)	moderate α-glucosidase inhibition, IC_50_ = 5.0 mM	-	[27]
	*R. glutinosa*; isolated compound	in vitro	LPS-treated rat NRK-52E cells	10 μM, 24 h; 0.1–100 μM, 72 h; EC_50_ = 15.3 μM	LPS control; no positive control	↑viability of LPS-injured cells: 1.043 ± 0.039 at 10 μM vs. 0.891 ± 0.043 model (*p* < 0.01); EC_50_ = 15.3 μM	-	[62]
frehmaglutin A (**73**)	*R. glutinosa*; isolated compound	in vitro	TGF-β1-treated BEAS-2B cells	10 μM	TGF-β1 control; no positive control	↑viability of TGF-β1-injured cells: 1.070 ± 0.011 vs. 1.000 ± 0.017 model (*p* < 0.05)	-	[61]
rehmaglutin A_1_ (**74**)	*R. glutinosa*; isolated compound	in vitro	TGF-β1-treated BEAS-2B cells	10 μM	TGF-β1 control; no positive control	↑viability of TGF-β1-injured cells: 1.115 ± 0.018 vs. 1.000 ± 0.017 model (*p* < 0.01)	-	[61]
frehmaglutoside I (**76**)	*R. glutinosa*; isolated compound	in vitro	DOX-treated rat H9c2 cardiomyocytes	40 and 80 μM, 24 h	DOX control; no positive control	↑viability of DOX-injured cardiomyocytes at 40 and 80 μM (*p* < 0.05 or *p* < 0.01 vs. DOX group)	-	[33]
*sec*-hydroxyaeginetic acid lactate (**79**)	*R. glutinosa*; isolated compound	in vitro	TGF-β1-treated BEAS-2B cells	10 μM	TGF-β1 control; no positive control	↑viability of TGF-β1-injured cells: 1.183 ± 0.011 vs. 1.000 ± 0.017 model (*p* < 0.01)	-	[61]
frehmaglutin C (**83**)	*R. glutinosa*; isolated compound	in vitro	D-galactosamine-treated human HL-7702 hepatocytes	10 μM, 1 h pretreatment	D-GalN control; bicyclol (10 μM)	↑cell survival to 61.2 ± 5.1% of normal (*p* < 0.01) vs. 48.3 ± 3.5% D-GalN control; 25.0% inhibition of injury; only active isolate, comparable to bicyclol	-	[31]
dihydroxy-*β*-ionone (**84**)	*R. glutinosa*; isolated compound	in vitro	LPS-treated rat NRK-52E cells	10 μM, 24 h; 0.1–100 μM, 72 h; EC_50_ = 40.7 μM	LPS control; no positive control	↑viability of LPS-injured cells: 1.011 ± 0.015 at 10 μM vs. 0.891 ± 0.043 model (*p* < 0.01); EC_50_ = 40.7 μM	-	[62]
rehmaionoside A (**86**)	*R. glutinosa*; isolated compound	in vitro	*α*-glucosidase (cell-free enzyme assay)	IC_50_ = 3.1 mM	untreated control; acarbose (IC_50_ = 3.0 mM)	moderate *α*-glucosidase inhibition, IC_50_ = 3.1 mM	-	[27]
rehmaionoside C (**88**)	*R. glutinosa*; isolated compound	in vitro	*α*-glucosidase (cell-free enzyme assay)	IC_50_ = 6.3 mM	untreated control; acarbose (IC_50_ = 3.0 mM)	moderate *α*-glucosidase inhibition, IC_50_ = 6.3 mM	-	[27]
	*R. glutinosa*; isolated compound	in vivo; in vitro; in silico	female BALB/c mice, LPS-induced S-AKI/LPS-treated TCMK-1 renal tubular cells	10 mg/kg, 3 d (route not stated); 1 μM in vitro	LPS control; resveratrol (10 mg/kg) + ICI 182780 (0.5 mg/kg) as blocker	↓serum Cr, BUN, Kim-1; ↓IL-6, TNF-α, IL-1β, MCP-1; ↓ROS, MDA; ↑SOD, GSH-GPx; ↓apoptosis; ↑renal ERα/ERβ; ↓TLR4, caspase 11 (*p* < 0.05/0.01 vs. model); effects blocked by ICI 182780	ERα/ERβ binding (docking −8.9 and −9.6; ERβ preferred over GPR30) → suppression of TLR4–caspase 11–IL-1β pathway	[120]
	*R. glutinosa*; isolated compound	in vitro	TGF-β1-treated BEAS-2B cells	10 μM	TGF-β1 control; no positive control	↑viability of TGF-β1-injured cells: 1.062 ± 0.023 vs. 1.000 ± 0.017 model (*p* < 0.05)	-	[61]
trihydroxy-*β*-ionone (**90**)	*R. glutinosa*; isolated compound	in vitro	LPS-treated rat NRK-52E cells	10 μM, 24 h; 0.1–100 μM, 72 h; EC_50_ = 17.7 μM	LPS control; no positive control	↑viability of LPS-injured cells: 1.039 ± 0.036 at 10 μM vs. 0.891 ± 0.043 model (*p* < 0.01); EC_50_ = 17.7 μM	-	[62]
abscisic acid (**96**)	*R. glutinosa*; isolated compound	in vitro	TGF-β1-treated BEAS-2B cells	10 μM	TGF-β1 control; no positive control	↑viability of TGF-β1-injured cells: 1.112 ± 0.028 vs. 1.000 ± 0.017 model (*p* < 0.01)	-	[61]
frehmaglutin D (**101**)	*R. glutinosa*; isolated compound	in vivo; in vitro; in silico	female BALB/c mice, LPS-induced AKI/LPS-treated TCMK-1 renal tubular cells	10 mg/kg, 3 d (route not stated); 1 μM in vitro	LPS control; resveratrol (10 mg/kg) + ICI 182780 (0.5 mg/kg) as blocker	↓serum Cr, BUN, Kim-1; ↓IL-6, TNF-α, IL-1β, MCP-1; ↓ROS, MDA; ↑SOD, GSH-Px; ↓apoptosis; ↑renal ERα/ERβ; ↓TLR4, caspase 11 (*p* < 0.05/0.01 vs. model); effects blocked by ICI 182780	preferential ERα binding (docking −14.2) over ERβ (−13.3), no GPR30 → suppression of TLR4–caspase 11–IL-1β pathway	[120]
	*R. glutinosa*; isolated compound	in vitro	LPS-treated rat NRK-52E cells	10 μM, 24 h; 0.1–100 μM, 72 h; EC_50_ = 10.2 μM	LPS control; no positive control	↑viability of LPS-injured cells: 1.104 ± 0.029 at 10 μM vs. 0.891 ± 0.043 model (*p* < 0.01); EC_50_ = 10.2 μM	-	[62]
**Iridoid + Ionone**								
rehmaglutoside F (**104**)	*R. glutinosa*; isolated compound	in vitro	APAP-treated human HepG2 cells	10 μM, 48 h	APAP control; bicyclol (10 μM)	↑cell survival to 63.4 ± 0.8% of normal vs. 58.5 ± 3.7% APAP control (*p* < 0.05)	-	[109]
rehmaglutoside I (**107**)	*R. glutinosa*; isolated compound	in vitro	D-GalN-treated HL-7702 hepatocytes	10 μM, 1 h pretreatment	D-GalN control; bicyclol (10 μM)	↑cell survival to 68.6 ± 1.9% of normal (*p* < 0.01); 28.2% inhibition of D-GalN-induced injury	-	[22]
rehmaglutoside G (**109**)	*R. glutinosa*; isolated compound	in vitro	D-GalN-treated HL-7702 hepatocytes	10 μM, 1 h pretreatment	D-GalN control; bicyclol (10 μM)	↑cell survival to 73.2 ± 5.0% of normal (*p* < 0.01); 38.7% inhibition of D-GalN-induced injury	-	[22]
**Phenylethanoid glycosides**								
jionoside C (**113**)	*Clerodendrum infortunatum*; isolated compound	in vitro	Hs578T and MDA-MB-231 triple-negative breast cancer cells	IC_50_ = 85.3 ± 2.4 μM (Hs578T); 96.5 ± 1.5 μM (MDA-MB-231); colony/migration assays run at IC_50_	vehicle control; no positive control	moderate antiproliferative/cytotoxic effect, IC_50_ = 85.3 ± 2.4 μM (Hs578T) and 96.5 ± 1.5 μM (MDA-MB-231); at IC_50_ ↓colony formation 42% (Hs578T) and 43% (MDA-MB-231), ↓migration 43% and 37%	-	[121]
salidroside (**114**)	commercially purchased	in vitro	male C57BL/6J mice, LAD-ligation myocardial infarction	200 mg/kg, intragastric, from 1 h post-MI for 21 d	MI control; no positive control	↑survival (80% vs. 50%), ↑LVEF, ↑LVFS, ↓LVESd, ↓LVEDd, ↓HW/BW, ↓LW/BW, ↓infarct size, ↓fibrosis and scar thickness, ↓TNF-α, TGF-β1, IL-1β, ↓apoptotic index, ↓Bax, ↑Bcl-2, ↑VEGF/Akt/eNOS (all *p* < 0.05)	activation of VEGF–Akt–eNOS pro-angiogenic signaling; anti-inflammatory and anti-apoptotic (↑Bcl-2/Bax)	[122]
isoacteoside (**123**)	*Scutellaria indica*; isolated compound	in vitro	LPS-treated RAW 264.7 cells	1, 3, 10, and 30 μM; IC_50_ = 44.6 ± 4.2 μM	LPS control; L-NMMA (IC50 22.1 ± 0.1 μM)	↓LPS-induced NO production in RAW 264.7 cells, IC_50_ = 44.6 ± 4.2 μM, weaker than L-NMMA (22.1 ± 0.1 μM); non-cytotoxic below 100 μM (MTS)	-	[123]
	*Callicarpa dichotoma*; isolated compound	in vitro	glutamate-treated primary rat cortical neurons	0.1, 1.0, and 10.0 μM	glutamate control; MK-801 (10 μM), CNQX (10 μM)	28.3 ± 1.8% (0.1 μM, *p* < 0.05), 42.2 ± 2.4% (1 μM, *p* < 0.01) and 67.9 ± 4.7% (10 μM, *p* < 0.001) protection against glutamate-induced injury (MK-801 87.2 ± 2.1%, CNQX 62.0 ± 4.7% at 10 μM)	reduction of glutamate-induced oxidative stress	[124]
	commercially purchased	in vitro	H_2_O_2_-treated SH-SY5Y neuroblastoma cells	10 and 50 μM, 12 h pre-incubation	H_2_O_2_ control; no positive control	pre-protective (antioxidant) effect on H_2_O_2_-injured SH-SY5Y cells at 50 μM; moderate, non-significant at 10 μM; no effect on bEnd.3 cells	-	[63]
acteoside (**124**)	commercially purchased	clinical	patients with biopsy-proven IgA nephropathy (*n* = 21 combination vs. *n* = 11 valsartan alone; 16 healthy controls)	0.8 g, p.o., 6 months (with valsartan 80–160 mg/d)	valsartan alone (80–160 mg/d)	24 h urinary protein fell from 1.64 ± 0.62 to 0.11 ± 0.09 g/d, versus 0.31 ± 0.13 g/d under valsartan alone (*p* = 0.015); peripheral blood Th22 cells fell from 1.08 ± 0.83% to 0.85 ± 0.29% (*p* < 0.001)	suppression of CCL20/CCL22/CCL27-driven Th22 cell chemotaxis and proliferation, with reduced IL-1, IL-6, and TNF-α and lower mesangial TGF-β1	[125]
	*R. glutinosa*; isolated compound	in vitro	α-glucosidase inhibition assay (cell-free)	4, 20, 100, 200, and 500 μM; IC_50_ = 261.4–408.7 μM (pooled for compounds **124**, **128**, **136**, and **140**)	untreated control; acarbose (IC_50_ = 204.2 ± 19.9 μM)	moderate inhibition; inhibition 67.0–94.0% at 500 μM for compounds **124**, **128**, **136**, and **140**, IC_50_ = 261.4–408.7 μM; weaker than acarbose	-	[23]
	*Callicarpa dichotoma*; isolated compound	in vitro	glutamate-treated primary rat cortical neurons	0.1, 1.0, and 10.0 μM	glutamate control; MK-801 (10 μM), CNQX (10 μM)	20.8 ± 2.5% (0.1 μM, not significant), 39.3 ± 3.1% (1 μM, *p* < 0.01) and 62.9 ± 4.7% (10 μM, *p* < 0.001) protection against glutamate-induced injury (MK-801 87.2 ± 2.1%, CNQX 62.0 ± 4.7% at 10 μM)	reduction of glutamate-induced oxidative stress	[124]
	*Clerodendron trichotomum*; isolated compound	in vitro	rat plasma ACE (cell-free)	dose-dependent; IC_50_ = 373.3 ± 9.3 μg/mL	untreated control; captopril (IC_50_ = 20 nM)	↓angiotensin-converting enzyme activity in rat plasma dose-dependently, IC_50_ = 373.3 ± 9.3 μg/mL	ACE inhibition; aromatic hydroxyls chelating the active-site Zn^2+^	[126]
	*Leonurus japonicus*; isolated compound	in vitro	D-galactosamine-treated human HL-7702 hepatocytes	10 μM	D-GalN control; bicyclol	↑survival of D-galactosamine-injured HL-7702 hepatocytes to 66% vs. 55% control (*p* < 0.05); inhibition 24.4%, comparable to bicyclol (66%, 25.3%)	-	[127]
	commercially purchased	in vivo	male C57BL/6 mice, B16 melanoma lung metastasis	50 mg/kg i.p., every other day from 13 d before B16 injection until death	vehicle control; no positive control	↑mean survival of B16 melanoma-injected C57BL/6 mice to 63.3 ± 3.4 d vs. 52.1 ± 2.5 d in controls (*p* < 0.05); 2/10 treated mice survived 80 d	antioxidant/ROS scavenging, ↓NO synthesis and direct cytotoxicity (B16 IC_50_ ≈ 200 μM/125 μg/mL)	[128]
	*Verbascum macrurum*; isolated compound	in vivo	ERE-luciferase HeLa (ERα/ERβ), MCF-7, KS483, Ishikawa cells	10^−7^–10^−9^ M (ERE-luciferase); 10^−6^–10^−8^ M (MCF-7, KS483)	vehicle control; 17β-estradiol (10^−8^–10^−9^ M) + ICI 182780 (10^−8^ M) as blocker	↓Basal ERE-luciferase via ERα and ERβ (*p* < 0.001, 10^−7^–10^−9^ M); ↑IGFBP-3 in MCF-7 (10^−6^–10^−8^ M); ↑osteoblast ALP; ↓mineralization at 10^−6^–10^−7^ M; no effect on Ishikawa cells	ER-mediated antiestrogenic action, mainly via ERα	[129]
	commercially purchased	in vitro	H_2_O_2_-treated SH-SY5Y neuroblastoma cells	10 and 50 μM, 12 h pre-incubation	H_2_O_2_ control; no positive control	pre-protective (antioxidant) effect on H_2_O_2_-injured SH-SY5Y cells at 50 μM; moderate, non-significant at 10 μM; no effect on bEnd.3 cells	-	[63]
echinacoside (**125**)	*Callicarpa dichotoma*; isolated compound	in vitro	glutamate-treated primary rat cortical neurons	0.1, 1.0, and 10.0 μM	glutamate control; MK-801 (10 μM), CNQX (10 μM)	19.7 ± 2.2% (0.1 μM, not significant), 48.7 ± 3.2% (1 μM, *p* < 0.01) and 68.3 ± 2.6% (10 μM, *p* < 0.001) protection against glutamate-induced injury (MK-801 87.2 ± 2.1%, CNQX 62.0 ± 4.7% at 10 μM)	reduction of glutamate-induced oxidative stress	[124]
leucosceptoside A (**128**)	*R. glutinosa*; isolated compound	in vitro	α-glucosidase inhibition assay (cell-free)	4, 20, 100, 200, and 500 μM; IC_50_ = 261.4–408.7 μM (pooled for compounds **124**, **128**, **136**, and **140**)	untreated control; acarbose (IC_50_ = 204.2 ± 19.9 μM)	67.0–94.0% at 500 μM for compounds **124**, **128**, **136**, and **140**, IC_50_ 261.4–408.7 μM; weaker than acarbose	-	[23]
	*Clerodendron trichotomum*; isolated compound	in vitro	rat plasma ACE (cell-free)	dose-dependent; IC_50_ = 423.7 ± 18.8 μg/mL	untreated control; captopril (IC_50_ = 20 nM)	↓angiotensin-converting enzyme activity in rat plasma dose-dependently, IC_50_ = 423.7 ± 18.8 μg/mL	ACE inhibition; Zn^2+^ chelation by free phenolic hydroxyls	[126]
jionoside A_1_ (**129**)	-	in vitro	5-FU-treated female BALB/c mouse bone marrow cells/H_2_O_2_-treated SH-SY5Y cells	0, 0.1, 1, 10, 25, and 50 μM (bone marrow cells); 10 and 50 μM (SH-SY5Y)	untreated control; no positive control	dose-dependent ↑proliferation of 5-FU-induced mouse bone marrow cells (CCK-8); pre-protective (antioxidant) effect on H_2_O_2_-injured SH-SY5Y cells at 50 μM	-	[63]
2′-acetylacteoside (**131**)	*Callicarpa dichotoma*; isolated compound	in vitro	glutamate-treated primary rat cortical neurons	0.1, 1.0, and 10.0 μM	glutamate control; MK-801 (10 μM), CNQX (10 μM)	30.1 ± 3.3% (0.1 μM, *p* < 0.05), 43.5 ± 4.4% (1 μM, *p* < 0.01), and 73.5 ± 6.2% (10 μM, *p* < 0.001) protection against glutamate-induced injury—the most active of the ten phenylethanoid glycosides tested (MK-801 87.2 ± 2.1%, CNQX 62.0 ± 4.7% at 10 μM)	reduction of glutamate-induced oxidative stress (caffeoyl moiety)	[124]
isomartynoside (**133**)	*Clerodendron trichotomum*; isolated compound	in vitro	rat plasma ACE (cell-free)	dose-dependent; IC_50_ = 505 ± 26.7 µg/mL	untreated control; captopril (IC_50_ = 20 nM)	↓angiotensin-converting enzyme activity in rat plasma dose-dependently, IC_50_ = 505 ± 26.7 μg/mL	ACE inhibition; fewest free aromatic hydroxyls, so weakest Zn^2+^ chelation	[126]
jionoside D (**134**)	*Clerodendron trichotomum*; isolated compound	in vitro	H_2_O_2_-treated V79-4 hamster lung fibroblasts	0.1, 1, and 10 μg/mL (1 h pre-treatment before 1 mM H_2_O_2_)	H_2_O_2_ control; N-acetylcysteine	↓intracellular ROS 41/62/85%, ↓DPPH 25/36/55%, ↓lipid peroxidation 11/22/28% at 0.1/1/10 μg/mL; ↑V79-4 survival 11/34/70%; ↓sub-G1 26%→16%; ↑SOD 12→39 and CAT 11→35 U/mg	ROS/DPPH radical scavenging with ↑SOD and catalase activity, ↓H_2_O_2_-induced apoptosis	[130]
martynoside (**135**)	*Clerodendron trichotomum*; isolated compound	in vitro	rat plasma ACE (cell-free)	dose-dependent; IC_50_ = 524.4 ± 28.1 μg/mL	untreated control; captopril (IC_50_ = 20 nM)	↓angiotensin-converting enzyme activity in rat plasma dose-dependently, IC_50_ = 524.4 ± 28.1 μg/mL	ACE inhibition; fewest free aromatic hydroxyls, so weakest Zn^2+^ chelation	[126]
	*Verbascum macrurum*; isolated compound	in vitro	ERE-luciferase HeLa (ERα/ERβ), MCF-7, KS483, Ishikawa cells	10^−7^–10^−9^ M (ERE-luciferase); 10^−6^–10^−9^ M (MCF-7, KS483); 0.16, 0.32, and 16 μM (Ishikawa)	vehicle control; 17β-estradiol + ICI 182780 (10^−7^–10^−8^ M) as blocker	↓basal ERE-luciferase via ERα and ERβ (*p* < 0.001); ↑IGFBP-3 in MCF-7; ↑ALP (10^−8^–10^−9^ M) and ↑mineralization to ~230% of control (10^−8^–10^−7^ M, abolished by ICI 182780); ↓Ishikawa proliferation (*p* < 0.05)	ER-mediated SERM-like action (ERα antagonism; ER-dependent osteoblast stimulation)	[129]
jionoside B_1_ (**136**)	*R. glutinosa*; isolated compound	in vitro	α-glucosidase inhibition assay (cell-free)	4, 20, 100, 200, and 500 μM; IC_50_ = 261.4–408.7 μM (pooled for compounds **124**, **128**, **136**, and **140**)	untreated control; acarbose (IC_50_ = 204.2 ± 19.9 μM)	α-glucosidase inhibition 67.0–94.0% at 500 μM for compounds **124**, **128**, **136**, and **140**, IC_50_ = 261.4–408.7 μM	-	[23]
brachynoside (**140**)	*R. glutinosa*; isolated compound	in vitro	α-glucosidase inhibition assay (cell-free)	4, 20, 100, 200, and 500 μM; IC_50_ = 261.4–408.7 μM (pooled for compounds **124**, **128**, **136**, and **140**)	untreated control; acarbose (IC_50_ = 204.2 ± 19.9 μM)	α-glucosidase inhibition 67.0–94.0% at 500 μM for compounds **124**, **128**, **136**, and **140**, IC_50_ = 261.4–408.7 μM	-	[23]
**Phenylpropanoids and their glycosides**								
nepetoidin F (**145**)	*R. glutinosa*; isolated compound	in vitro	LPS-treated NRK-52E rat kidney tubular epithelial cells	10 μM, 24 h	LPS control; no positive control	protection against LPS-induced NRK-52E injury; cell viability 0.9910 ± 0.0447 vs. 0.8418 ± 0.0389 model (normal control 1.0000 ± 0.0617)	-	[49]
jiofuran acid A (**146**)	*R. glutinosa*; isolated compound	in vitro	TGF-β1-treated BEAS-2B cells	10 μM, 24 h	TGF-β1 control; no positive control	↑viability of TGF-β1-treated BEAS-2B cells to 1.054 ± 0.004 vs. 1.000 ± 0.007 model (*p* < 0.05)	-	[66]
**Flavonoids**								
apigenin (**147**)	commercially purchased	in vivo	male Swiss albino mice, alloxan-induced type 1 diabetes	0.78 mg/kg, s.c., 10 d	diabetic control; no positive control	↓serum glucose (297.7→73.34 mg/dL), cholesterol (68.36→39.77 mg/dL), hepatic G-6-Pase (4.38→1.86) and LPO (2.92→0.95); ↑SOD (2.51→5.37), CAT (30.58→45.72), GSH (1.27→6.87), insulin, T3, T4	↑insulin and thyroid hormone secretion; ↓hepatic gluconeogenic G-6-Pase; antioxidant (SOD/CAT/GSH) restoration	[131]
	commercially purchased	in vivo	male C57BL/6J mice, STZ-induced diabetic cardiomyopathy	100 mg/kg/d, p.o., 7 months	diabetic control; no positive control	↑EF (63.60→70.67%) and FS (35.78→38.92%); ↓blood glucose (26.09→24.00 mmol/L); ↓cardiomyocyte CSA, ANP/BNP, fibrosis, 4-HNE, MDA, Bax, cleaved caspase-3; ↑GPx, SOD, Bcl-2	inhibition of IκBα phosphorylation and NF-κB/p65 nuclear translocation; ↓TNF-α, IL-1β, IL-6; anti-oxidative and anti-apoptotic	[132]
	commercially purchased	in vitro	high-glucose-treated H9c2 cardiomyocytes	25 μM, 12–72 h (high-glucose 4.5 g/L DMEM)	high-glucose control; resveratrol (25 μM)	↓ROS production, ↓Bax, ↑Bcl-2, ↓NF-κB/p65 nuclear translocation in high-glucose H9c2 cells; effect equal to resveratrol 25 μM	blockade of high-glucose-induced NF-κB/p65 nuclear translocation; anti-oxidative and anti-apoptotic (Bax/Bcl-2)	[132]
	laboratory-prepared (source not stated)	in vivo; in vitro	male Sprague–Dawley rats, HFD/STZ type 2 diabetes/palmitic acid-treated HUVECs	50 and 100 mg/kg/d, intragastric, 6 w; 3 and 30 μM (HUVECs)	diabetic control; metformin (160 mg/kg/d)	↓fasting blood glucose (26.27→8.25 and 7.22 mmol/L), glucose AUC (72.22→46.60 and 47.37 mmol/L·h), TC, TG, LDL-C, FFA, GSP, MDA (10.94→5.49 and 5.14 nmol/L); ↑HDL-C, SOD; ↓ICAM-1; aortic ACh Emax 31.7→98.7 and 97.7%	↓NF-κB activation and ICAM-1 mRNA expression; ↓oxidative stress; restoration of insulin-mediated endothelial relaxation	[133]
luteolin (**148**)	commercially purchased	in vivo; in vitro	male Sprague–Dawley rats /LPS-treated murine RAW264.7 macrophages	20 mg/kg, p.o., 8 w; 25 μM for 24 h in RAW264.7 cells (0–50 μM viability range)	LPS control; luteolin-7-O-glucoside (20 mg/kg), luteolin glucuronides (25 μM)	luteolin absorbed mainly as luteolin-3′-O-glucuronide (plasma Cmax 4.1 μM at 3 h); 25 μM luteolin ↓RAW264.7 viability to ~65% and ↓LPS-induced IL-6, IL-1β, NF-κB1, Ccl2/3/5, JunB more strongly than its glucuronides	glucuronidation to luteolin-3′-O-glucuronide in vivo; suppression of LPS-induced inflammatory gene transcription (NF-κB pathway)	[134]
chrysoeriol (**149**)	*Cardiospermum halicacabum*; isolated compound	in vivo; in silico	male Wistar rats, STZ-induced diabetes	20 mg/kg, p.o., 45 d	diabetic control; glibenclamide (600 μg/kg)	↓plasma glucose (282.16→133.27 mg/dL), HbA1c (13.02→6.52 mg/g Hb), TC (153.63→90.26), TG (164.39→86.25), LDL-C, FFA, atherogenic index (6.44→2.15); ↑insulin (6.23→15.87 μU/mL), HDL-C, LPL, LCAT	↓hepatic HMG-CoA reductase and ↑LPL/LCAT activity; docking to HMG-CoA reductase (−7.59), LPL (−7.82), and LCAT (−6.94) kcal/mol	[135]
***Lignans***								
(+)-paulownin (**156**)	*R. glutinosa*; isolated compound	in vitro	TGF-β1-treated BEAS-2B cells	10 μM, 24 h	TGF-β1 control; no positive control	↑viability of TGF-β1-treated BEAS-2B cells to 1.083 ± 0.038 vs. 1.000 ± 0.007 model (*p* < 0.01; normal control 1.221 ± 0.003)	-	[66]
8′-hydroxyl episesaminone (**157**)	*R. glutinosa*; isolated compound	in vitro	TGF-β1-treated BEAS-2B cells	10 μM, 24 h	TGF-β1 control; no positive control	↑viability of TGF-β1-treated BEAS-2B cells to 1.101 ± 0.007 vs. 1.000 ± 0.007 model (*p* < 0.01)	-	[66]
rhemaneolignan A (**163**)	*R. glutinosa*; isolated compound	in vitro	DOX-treated rat H9c2 cardiomyocytes	40 and 80 μM, 24 h	DOX control; no positive control	↑viability of DOX-injured cardiomyocytes at 40 and 80 μM (*p* < 0.05 or *p* < 0.01 vs. DOX group)	-	[33]
rhemaneolignan B (**164**)	*R. glutinosa*; isolated compound	in vitro	DOX-treated rat H9c2 cardiomyocytes	40 and 80 μM, 24 h	DOX control; no positive control	↑viability of DOX-injured H9c2 cardiomyocytes at 40 and 80 μM; *p* < 0.05 or *p* < 0.01 vs. DOX group	-	[33]
**Terpenoids**								
rehmapicrogenin (**173**)	commercially purchased	in vivo	male C57BL/6 mice, DOX-induced nephropathy	25 and 50 mg/kg, p.o., 7 d	DOX control; no positive control	↓Scr 242.2→69.9/56.6, ↓BUN 16.8→4.3/6.4, ↓24 h urine protein 495.9→262.0/235.8 mg/L, ↑U-ALB, ↓ROS 98.3→43.9/31.8, ↓MDA, ↑SOD, ↑GSH-Px	Nrf2/ARE activation (↑Nrf2, KEAP1, HO-1, HDAC2, p-PKC) via estrogen receptor (ERα/ERβ/GPR30) signaling	[136]
	commercially purchased	in vitro	DOX-treated rat NRK-52E cells	1, 5, and 10 μmol/L for 24 h (5 μmol/L in mechanistic assays)	DOX control; no positive control; ICI 182780 (fulvestrant, 1 μg/L) as ER blocker	↑viability 0.72→1.04 (5 μmol/L), ↓apoptosis 34.6%→9.3% (5 μmol/L) and 13.0% (10 μmol/L), ↑Nrf2, KEAP1, HO-1, HDAC2; effects abolished by ICI 182780	estrogen receptor-dependent Nrf2/KEAP1/HO-1/HDAC2 activation, ↓ROS	[136]
	*R. glutinosa*; isolated compound	in vitro	TGF-β1-treated BEAS-2B cells	10 μM	TGF-β1 control; no positive control	↑viability of TGF-β1-injured cells: 1.092 ± 0.034 vs. 1.000 ± 0.017 model (*p* < 0.05)	-	[61]
	commercially purchased	in vitro	LPS-treated MPC5 podocytes	5 and 10 μmol/L, 2 h pretreatment before LPS (48 h)	LPS control; no positive control + ML385 (10 μM) as blocker	↑cell viability, ↓apoptotic rate, ↑Bcl-2, ↓Bax and cleaved caspase-3, ↑nuclear Nrf2 and HO-1, ↓Keap1; all reversed by ML385—no numeric values reported	activation of Nrf2/ARE signaling, ↓apoptosis and oxidative stress	[137]
	commercially purchased	in vivo	male C57BL/6 mice, LPS-induced acute kidney injury	25 and 50 mg/kg, p.o., every 6 h from 1 h after LPS until sacrifice at 24 h	LPS control; no positive control	dose-dependent ↑body weight and urine volume, ↓24 h urine protein, ↓creatinine, ↓BUN, ↑urinary albumin, ↓TUNEL^+^ podocytes, ↑nephrin/podocin, ↓MDA, ↑SOD	activation of Nrf2/ARE signaling (↑Nrf2, HO-1, HDAC2, p-PKC) in podocytes	[137]
rehmapicroside (**174**)	commercially purchased	in vivo	adult male Sprague–Dawley rats, transient MCAO cerebral ischemia	2.5, 5, and 10 mg/kg, single i.p. injection at onset of reperfusion (effects significant at 10 mg/kg)	MCAO control; FeTMPyP (3 mg/kg)	neuroprotective activity: ↓Infarct size and ↓mNSS scores at 10 mg/kg (comparable to FeTMPyP 3 mg/kg), ↓3-nitrotyrosine, ↓Drp1 nitration, ↓NOX/iNOS, ↓mitochondrial PINK1/Parkin/Drp1 translocation	direct ONOO^−^ scavenging; inhibition of ONOO^−^-mediated PINK1/Parkin/Drp1 mitophagy	[138]
rehmapicrogenin monomethyl ester (**178**)	*R. glutinosa*; isolated compound	in vivo	TGF-β1-treated BEAS-2B cells	10 μM	TGF-β1 control; no positive control	↑viability of TGF-β1-injured cells: 1.104 ± 0.004 vs. 1.000 ± 0.017 model (*p* < 0.05)	-	[61]
cedrol (**179**)	commercially purchased	in vivo	male C57BL/6J mice, HFD obesity	0.2% (*w*/*w*) in HFD for 19 w	HFD control; no positive control	↓body-weight gain, ↓visceral fat pad weight, ↓adipocyte size, ↓liver weight and steatosis, ↓ALT 268.7→94.2 U/L, ↓AST 210.7→153.1, ↓TC 7.94→3.57, ↓LDL-C 1.58→0.54, ↓MDA 62.8→12.2 nmol/mL, ↓AUC of OGTT/ITT/PTT	↓hepatic gluconeogenesis, improved insulin sensitivity; transcriptomic reprogramming of epididymal WAT fatty-acid metabolism genes (Cyp2e1, Elovl2, Echdc2, Acsm3); minimal gut microbiota change	[139]
	-	in vivo	male Wistar rats, LPS-induced liver/kidney injury	7.5, 15, and 30 mg/kg, p.o., 30 min before LPS, 2 w	LPS control; no positive control	30 mg/kg ↓TNF-α and ↓MDA in liver/kidney, ↓urea, ↑total thiol, ↑SOD and CAT; 15 and 30 mg/kg ↓AST, ALT, ALK-P, and creatinine	anti-inflammatory and antioxidant action (↓TNF-α, ↓lipid peroxidation, ↑thiol/SOD/CAT)	[140]
	commercially purchased	in vivo	male C57BL/6 mice, MCAO cerebral ischemia	40 mg/kg, p.o., 1 and 2 days after reperfusion (3 d)	MCAO control; no positive control	↓infarct size and ↓mNSS at d 3, ↑grip strength, ↓grid-walking foot faults, ↑rotarod latency, ↓IL-1β, IL-6, TNF-α, and COX-2 in penumbra, ↓p-IκB and ↓p-P65	ERβ binding (docking −4.43 kcal/mol) with inhibition of NF-κB signaling in microglia; blocked by the ER antagonist fulvestrant	[141]
	*Platycladus orientalis*; isolated compound	in vivo	male C3H/HeJ mice, imiquimod-induced alopecia areata	30 mg/kg, p.o., 28 d (in 0.5% CMC-Na or corn oil)	imiquimod control; baricitinib (1 mg/kg)	↑hair length at 25 d 3.97→6.14 mm vs. baricitinib 5.68 mm; ↑average hair weight 18.35→28.2 mg (baricitinib 21.08); ↑Ki67^+^ cells; ↓IL-1β, IL-6, IL-33, CXCL13/18/24; normalized spleen index	rebalancing of M1/M2 macrophage polarization and ↓IFN-γ-driven inflammation via STAT and MAPK (p38/JNK) signaling	[142]
	*Juniperus chinensis*; isolated compound	in vitro	HT29 colorectal carcinoma cells	20, 25, and 30 μg/mL for 24–48 h	vehicle control; no positive control	↓HT29 growth by 33.64%, 57.51%, and 68.82% at 20, 25, and 30 μg/mL (48 h); G1 phase 56.36%→73.26%; apoptosis 9.12%→32.68%; ↓MCM proteins, PCNA, CDK2, cyclin E, pRb; ↑p53, p21	downregulation of minichromosome maintenance (MCM) proteins → G1 arrest; caspase-3/-8/-9 activation and PARP cleavage with cytochrome-c release	[143]
	*Juniperus chinensis*; isolated compound	in vivo	female BALB/c-nu athymic mice, HT29 colorectal xenograft	50 mg/kg, i.v., three times weekly for 14 d	vehicle control; DOX (3 mg/kg)	↓tumor volume and weight, more effective than DOX 3 mg/kg; normal body weight maintained (weight loss in DOX group); ↓tumor cell density and ↓vascularization, ↓MCM7 immunostaining	MCM7 downregulation in tumor tissue with reduced angiogenesis	[143]
remophilanetriol F (**187**)	*R. glutinosa*; isolated compound	in vitro	TGF-β1-treated BEAS-2B bronchial epithelial cells	10 μM, 24 h	TGF-β1 control; no positive control	↑viability of TGF-β1-injured cells: 1.091 ± 0.018 vs. 1.000 ± 0.012 model (*p* < 0.01); ↓intracellular ROS and apoptosis rate (*p* < 0.05 or *p* < 0.01)	↓TGF-β1-induced oxidative stress and apoptosis; no molecular pathway identified	[19]
remophilanetriol A (**188**)	*R. glutinosa*; isolated compound	in vitro	LPS-treated NRK-52E rat renal tubular cells	10 μM, 24 h	LPS control; no positive control	MTT: ↑viability of LPS-injured NRK-52E cells to 88.56 ± 5.07% vs. 69.08 ± 9.47% for LPS alone (*p* < 0.01; control 100 ± 12.03%)	-	[45]
remophilanetriol B (**190**)	*R. glutinosa*; isolated compound	in vitro	LPS-treated NRK-52E rat renal tubular cells	10 μM, 24 h	LPS control; no positive control	MTT: ↑viability of LPS-injured NRK-52E cells to 86.41 ± 5.17% vs. 69.08 ± 9.47% for LPS alone (*p* < 0.01; control 100 ± 12.03%)	-	[45]
glutinosalactone C (**208**)	*R. glutinosa*; isolated compound	in vitro	MCF-7, MG63, and HepG2 human cancer cells	0, 1, 10, 25, 50, and 100 μM, 72 h; IC_50_ = 8.35 ± 0.16 μM (MCF-7), 39.25 ± 0.21 μM (MG63), and 17.29 ± 2.16 μM (HepG2)	untreated control; no positive control	cytotoxic to all three lines; IC_50_ = 8.35 ± 0.16 μM (MCF-7), 39.25 ± 0.21 μM (MG63), and 17.29 ± 2.16 μM (HepG2)	-	[46]
*β*-sitosterol (**209**)	commercially purchased	in vivo; in silico	male Wistar rats, HFD/sucrose type-2 diabetes	20 mg/kg, p.o., 30 d	diabetic control; metformin (50 mg/kg/d)	↓p-IRS-1Ser636 (*p* = 0.0003); ↑IR (*p* = 0.0036), IRS-1, β-arrestin-2, Akt, AS160, GLUT4 mRNA; ↑p-IRS-1Tyr632 (*p* = 0.0014), p-AktSer473/Thr308; docking ΔGbind −70.67 to −49.01 kcal/mol	IRS-1/Akt-mediated insulin signaling; β-arrestin-2/c-Src scaffolding; AS160/GLUT4 trafficking	[144]
daucosterol (**210**)	-	in vitro	OGD/R-injured primary rat cortical neurons (E18 Sprague–Dawley)	10 and 20 μM for 24 h post-OGD/R (screened 1.25–100 μM)	OGD/R control; no positive control + picropodophyllin (200 nM) as blocker	↑neuronal viability at 20 μM (*p* < 0.01) and 10 μM (*p* < 0.05); ↓caspase-3 activity, ↓apoptotic rate (*p* < 0.001); ↑IGF1 (*p* < 0.001), ↑p-AKT, p-GSK-3β, Mcl-1, Bcl-2, Bcl-2/Bax; ↓Bax	IGF1/IGF1R–AKT/GSK-3β activation with Bcl-2/Bax-mediated anti-apoptosis	[145]
	commercially purchased	in vitro	MGC803, BGC823, AGS, and MCF-7 cancer cells	IC_50_ = 19.96 μM (MGC803), 3.13 μM (BGC823), 24.19 μM (AGS), and 16.95 μM (MCF-7); 50 μM used in mechanistic assays	vehicle control; no positive control	↓proliferation of MGC803, BGC823, AGS and MCF-7 cells dose-dependently (IC_50_ = 3.13–24.19 μM); ↓colony formation; ↑ROS 1.48-fold (MCF-7) and 2.25-fold (BGC823); ↑LC3-II, no apoptosis	ROS-dependent autophagy induction (LC3-II conversion); reversed by GSH/NAC and 3-MA	[146]
	commercially purchased	in vivo	ICR mice, H22 hepatoma allograft	0.5 and 2.5 mg/kg, p.o., 7 d	vehicle control; cyclophosphamide	↓tumor weight to 0.71 ± 0.29 g at 0.5 mg/kg (*p* < 0.01) and 0.46 ± 0.28 g at 2.5 mg/kg vs. 1.51 ± 0.23 g control; no body-weight loss; no lesions in heart, liver or kidney	ROS-dependent autophagy (inferred from in vitro data); no in vivo mechanistic assay	[146]
	*Astragalus membranaceus*; isolated compound	in vivo; in vitro	*C. albicans* (in vitro susceptibility)/BALB/c female mice, disseminated candidiasis	200 μL of 200 μg/mL (40 μg/mouse), i.p., twice at a 3-day interval before challenge	vehicle control; amphotericin B	↑mean survival 28.8 ± 10.7 ds vs. all 5 controls dead by d 12 (*p* < 0.01); ↓kidney C. albicans CFU ≈81% (*p* < 0.01); ↑IFN-γ and IL-2 over IL-4/IL-10; no direct growth inhibition in vitro	CD4+ Th1 polarization with IFN-γ/IL-2 production; abolished by anti-CD4 or anti-IFN-γ, transferable by CD4+ T cells	[147]
**Miscellaneous constituents**								
5-hydroxymethyl-2-furaldehyde (**235**)	commercially purchased	in vitro	corticosterone-treated primary rat hippocampal neurons (newborn Sprague–Dawley)	0.5 mg/L for 24 h	corticosterone control; donepezil (40 μmol/L) + RU38486 (3 nmol/L) as blocker	↑cell vitality 0.615 ± 0.021 vs. model 0.538 ± 0.041; ↓β-galactosidase 0.353 ± 0.051 vs. 0.464 ± 0.040; ↑GCR 0.193 ± 0.027, BDNF 0.055 ± 0.009, NR2B 0.773 ± 0.069, p-ERK 0.605 ± 0.147, p-CREB 0.582 ± 0.104, p-synapsin 0.213 ± 0.045 (all *p* < 0.05 vs. model)	restoration of the GCR–BDNF–NR2B–p-ERK–p-CREB–p-synapsin signal transduction pathway	[148]
	commercially purchased	in vivo; in vitro	male C57BL/6J mice, DMM-induced osteoarthritis/IL-1β-treated ATDC5 chondrocytes	7.5, 15, and 30 mg/kg, i.p., 8 w (in vivo); 10 μg/mL with IL-1β for 24 h (chondrocytes)	DMM control; celecoxib (26 mg/kg/d)	↓subchondral BV/TV and Tb.Th, ↑cartilage area, ↓OARSI score, ↑Col2a1, ↓MMP13, ↑Glut1/HK1/LDHA—comparable to celecoxib; benefit lost in Glut1AgcER chondrocyte-knockout mice	upregulation of chondrocyte glucose metabolism via the Glut1/HK1/LDHA axis	[149]
rehmanniaeketone C (**242**)	*R. glutinosa*; isolated compound	in vitro	human A549 lung carcinoma cells	IC_50_ = 2.05 μM	untreated control; no positive control	strongest of the five isolates against A549 lung cancer cells, IC_50_ = 2.05 μM; tested concentration range not reported by the authors	-	[74]
rehmanniaeketone B (**244**)	*R. glutinosa*; isolated compound	in vitro	human A549 lung carcinoma cells	IC_50_ = 5.23 μM	untreated control; no positive control	inhibitory activity against A549 lung cancer cells, IC_50_ = 5.23 μM; tested concentration range not reported by the authors	-	[74]
2,5-dihydroxyacetophenone (**252**)	commercially purchased	in vivo	male Sprague–Dawley rats, CLP-induced septic AKI	40 mg/kg (low) or 80 mg/kg (high), p.o., 2 h after CLP surgery	CLP control; no positive control	↑survival >50% at 72 h vs. 10% at 72 h in CLP rats; ↓serum LDH, lactate, Scr, BUN, cystatin C, NGAL, KIM-1; ↓renal TNF-α, IL-1β, TGF-β1 mRNA; ↓renal injury score; ↑renal artery blood flow	inhibition of ERK1/2 phosphorylation and nuclear NF-κB p65 translocation	[150]
2,3,5,4′-tetrahydroxystilbene-2-*O*-*β*-D-glucoside (**263**)	*R. glutinosa*; isolated compound	in vitro	yeast α-glucosidase (cell-free)/H_2_O_2_-treated INS-1 rat insulinoma cells	IC_50_ = 0.186 ± 0.003 μmol/mL (α-glucosidase); 0, 40, and 200 μg/mL (kinetics); 5, 10, 20, 40, and 80 μM (cells)	untreated control; acarbose (IC_50_ = 5.42 ± 0.09 μg/mL), vitamin C	non-competitive reversible α-glucosidase inhibition, IC_50_ = 0.186 ± 0.003 μmol/mL, Km 5.23 mmol/L unchanged, ↓Vmax; ↑INS-1 viability 52.13% to 55.42, 57.19, 61.18, 65.24, 76.26% at 5–80 μM; ↓MDA 213.68% to 160.00, 103.16, 67.47%; ↑SOD 298.34 U/mg; ↑insulin 1.47 ± 0.113 ng/mL (+59.37%, 91.29% of normal, +22.02% total secretion)	α-glucosidase inhibition plus Nrf2/HO-1-mediated antioxidant protection of β cells	[75]
3,8-dihydroxy-2-(2-hydroxyethyl)chromone (**264**)	*R. glutinosa*; isolated compound	in vitro	human A549 lung carcinoma cells	IC_50_ = 61 μM	untreated control; no positive control	moderate inhibitory activity against A549 lung cancer cells, IC_50_ = 61.0 μM; tested concentration range not reported by the authors	-	[74]
nicotinic acid (**271**)	*R. glutinosa*; isolated compound	in vitro	LPS-treated NRK-52E rat kidney tubular epithelial cells	10 μM, 24 h	LPS control; no positive control	highest protection of the series against LPS-induced NRK-52E injury; cell viability 1.0502 ± 0.0743 vs. 0.8418 ± 0.0389 model (normal control 1.0000 ± 0.0617)	-	[49]
[rehmagluamide (**304**)	*R. glutinosa*; isolated compound	in vitro	LPS-treated NRK-52E rat kidney tubular epithelial cells	10 μM, 24 h	LPS control; no positive control	protection against LPS-induced NRK-52E injury; cell viability 1.0126 ± 0.1277 vs. 0.8418 ± 0.0389 model (normal control 1.0000 ± 0.0617)	-	[49]
oleic acid (**313**)	*R. glutinosa*; isolated compound	in vivo; in vitro	LPS-treated NRK-52E renal tubular cells/male BALB/c mice, LPS-induced acute kidney injury	10 and 30 mg/kg in vivo (route and duration not stated); 10 μM in vitro	LPS control; no positive control + FTS as blocker	↓serum Cr, BUN, renal histopathology; ↓IL-2, TNF, IFN-γ, ↑IL-10; ↓iNOS, COX-2, p-p65, ROS, MDA; ↑GSH-Px, T-SOD, p-Nrf2, HO-1, Bcl-2; ↓cleaved caspase-9/-3, Bax; in NRK-52E ↑Ras, Raf1, SHC, PPAR-γ, reversed by FTS	Ras/MAPKs/PPAR-γ signaling with Keap1/Nrf2/HO-1 activation and NF-κB p65 inhibition	[151]
Extracts								
steamed roots; water extract (freeze-dried granules, 0.52% 5-HMF by HPLC)	*R. glutinosa* roots	clinical	Korean women with obesity, BMI ≥ 25 kg/m^2^, aged 40–65 y; open-label single-arm trial (*n* = 12; KCT0000650)	4 g granules twice daily, p.o., 8 w	none (before–after comparison)	waist circumference fell from 947.3 ± 67.92 to 922.4 ± 74.64 mm (*p* = 0.035); body weight (71.5 ± 7.88 → 70.6 ± 7.95 kg, *p* = 0.125), BMI (*p* = 0.126), body fat (*p* = 0.905), fasting glucose (*p* = 0.791), and lipids were unchanged; fecal *Actinobacteria* increased (*p* = 0.047) with *Bifidobacterium* replacing *Blautia* as the predominant genus	prebiotic modulation of the gut microbiota by the oligosaccharide-rich extract	[152]
dried roots; boiling-water decoction (catalpol 44.85 ± 0.85 mg/g, rehmannioside D 4.38 ± 0.13 mg/g, ajugol 5.50 ± 0.15 mg/g, and aucubin 0.57 ± 0.02 mg/g by HPLC)	*R. glutinosa* roots	in vivo	carrageenan-induced tail thrombosis in male Sprague–Dawley rats (200 ± 20 g, *n* = 10 per group)	1.35, 2.7, and 5.4 g/kg, p.o., 7 d before and 2 days after carrageenan	model control; enteric-coated aspirin (20 mg/kg)	thrombus length shortened at every time point at 5.4 g/kg (*p* < 0.01) and at 24 and 48 h at 2.7 g/kg (*p* < 0.01) but not at 1.35 g/kg; APTT and TT prolonged and fibrinogen reduced at 5.4 g/kg (*p* < 0.01); the serum ET-1/NO ratio, TNF-α, and IL-6 fell at all three doses (*p* < 0.01); no histopathological vascular damage at 5.4 g/kg	suppression of glycerophospholipid → arachidonic acid → thromboxane A2 flux; docking to PLA2G2A, PTGS1, and ALOX5	[153]
fresh tubers, freeze-dried; water extract (catalpol 57.13 ± 1.40 mg/g, verbascoside 28.97 ± 4.50 mg/g, isoacteoside 21.38 ± 1.43 mg/g, rehmannioside D 3.80 ± 0.93 mg/g, and ajugol 2.80 ± 0.19 mg/g by UPLC-DAD)	*R. glutinosa* tubers	in vivo	HFD/STZ (35 mg/kg, i.p.)-induced diabetic nephropathy in male Sprague–Dawley rats (*n* = 6 per group)	250 and 500 mg/kg, p.o., 4 w	diabetic nephropathy model control; metformin (200 mg/kg)	body weight regained by 18.24% at 500 mg/kg versus 10.98% for metformin; serum triglyceride, LDL-C, BUN, creatinine, AST, and ALT all reduced (*p* < 0.01) and HDL-C raised (*p* < 0.01); renal MDA lowered with SOD, CAT and GSH-Px restored; serum TNF-α, IL-1β, IL-6 and MCP-1 reduced at 500 mg/kg (*p* < 0.01); kidney organ index returned to normal	downregulation of TLR4/MyD88/NF-κB p65/TRAF6 and NOX-4 with upregulation of NQO1 and HO-1	[154]
dried leaves; 80% ethanol reflux (acteoside 6.833 mg/g, catalpol 6.326 mg/g, and ajugol 4.105 mg/g by UPLC-QTOF/MS)	*R. glutinosa* leaves	In vivo	spontaneously diabetic male db/db mice with early kidney injury (*n* = 8 per group; db/m mice as normal control)	2.6 g/kg, p.o., 6 w	db/db model control; metformin (250 mg/kg), irbesartan (50 mg/kg), and a marketed Dihuangye total glycoside capsule (520 mg/kg, acteoside-matched)	fasting glucose, insulin, total cholesterol, triglyceride, BUN, microalbuminuria, and creatinine all normalized (*p* < 0.05–0.001), with BUN and total cholesterol lowered further than by the three comparators; the *Firmicutes* and *Bacteroidetes* restored (model 53.8 ± 9.551 and 44 ± 9.695 versus control 74.98 ± 6.265 and 20.75 ± 6.492, *p* ≤ 0.0045) together with recovery of *Lachnospiraceae* NK4A136 and *Roseburia*	inhibition of TGF-β1/Smad3/Smad4-driven α-SMA expression, with restoration of gut microbiota and of ether lipid, sphingolipid, and arachidonic acid metabolism	[155]
steamed roots; 70% ethanol reflux (yield 26.82% *w*/*w*)	*R. glutinosa* roots	in vivo; in vitro	*Dermatophagoides farinae* extract-induced atopic dermatitis in male NC/Nga mice (*n* = 7–8 per group), topical treatment from d 11 for 23 ds; IFN-γ/TNF-α-stimulated HaCaT keratinocytes	400 μg/mouse, applied topically in acetone/olive oil (4:1)	Mite allergen control; 0.1% tacrolimus ointment (100 μg/mouse)	dermatitis score reduced from d 21 (*p* < 0.05) and ear swelling suppressed to a degree not different from tacrolimus; serum histamine reduced (*p* < 0.05); lesional IL-4, TNF-α, TARC, MDC, RANTES, ICAM-1, and VCAM-1 mRNA all reduced (*p* < 0.05); in IFN-γ/TNF-α-stimulated HaCaT keratinocytes, the 14-fold rise in TARC and the 6- and 7-fold rises in MDC and RANTES were inhibited dose-dependently up to 200 μg/mL without cytotoxicity; serum IgE was not reduced but rose, as with tacrolimus	keratinocyte-level suppression of TARC/MDC/RANTES and of ICAM-1 and VCAM-1, limiting Th2 cell and eosinophil recruitment; independent of serum IgE	[156]
steamed roots; aqueous extract (RRP_AE_) and its glycoside (RRP_GLY_), polysaccharide (RRP_POL_), and organic acid (RRP_OA_) fractions	Radix Rehmanniae Praeparata	in vivo	hepatic ischemia–reperfusion injury in male C57BL/6J mice (8 w, 22–24 g, *n* = 6 per group), 1 week of pretreatment	RRP_AE_ 5 g/kg, RRP_GLY_ 1 g/kg, RRP_POL_ 190 mg/kg, and RRP_OA_ 120 mg/kg, p.o., 7 days before surgery	ischemia–reperfusion model control; *N*-acetylcysteine (100 mg/kg)	serum ALT, AST, and LDH reduced and nitric oxide restored by the aqueous extract and by the glycoside and polysaccharide fractions; the glycoside fraction was the most active, restoring sinusoidal fenestrae and FITC-formaldehyde-serum-albumin uptake and lowering SA-β-gal, p53, and p21 together with the senescence-associated secretory genes Il-1b, Cxcr2, Timp1, and Mmp13; the organic acid fraction gave no protection and showed a trend towards worse senescence markers	reversal of LRP1–NOTCH1–C/EBPβ-mediated senescence of liver sinusoidal endothelial cells, with loss of C/EBPβ occupancy at the *Il-1b* promoter	[157]

↑, increase or upregulation; ↓, decrease or downregulation; →, mechanistic or sequential relationship. * *p* < 0.05, ** *p* < 0.01 vs.

Apigenin (**147**) exhibited antidiabetic activity in alloxan-induced diabetic Swiss albino mice [131]. Subcutaneous administration of apigenin (0.78 mg/kg/d for 10 ds) restored the levels of insulin and thyroid hormone (T3 and T4), while reducing serum glucose, hepatic glucose-6-phosphatase activity, and cholesterol. Compound **147** also attenuated oxidative stress by suppressing hepatic lipid peroxidation and restoring the levels of catalase (CAT) SOD, and glutathione (GSH), thereby exerting hepatoprotective effects.

Apigenin (**147**, 50 or 100 mg/kg, 6 w) improved glucose homeostasis and endothelial function in HFD/STZ-induced type 2 diabetic rats, with metformin (160 mg/kg/d) as the positive control [133]. Treatment markedly reduced fasting blood glucose (26.27 to 7.22–8.25 mmol/L) and improved glucose tolerance, thus lowering AUC of glucose (72.22 to 46.60–47.37 mmol/L·h). In addition, **147** reduced MDA and intercellular adhesion molecule-1 (ICAM-1) levels and increased SOD activity. Vascular dysfunction was alleviated through restoration of contraction and relaxation. In palmitic acid-treated human umbilical vein endothelial cells (HUVECs), apigenin (3 or 30 μM) suppressed nuclear factor-κB (NF-κB) activation and ICAM-1 mRNA expression and restored insulin-stimulated NO production, suggesting that the protective effects of **147** are associated with attenuation of oxidative stress and inflammation.

Apigenin (**147**, 100 mg/kg/d) attenuated diabetic cardiomyopathy in STZ-induced diabetic mice [132]. Apigenin treatment improved cardiac function, as evidenced by increased ejection fraction and fractional shortening, while reducing ventricular hypertrophy, cardiomyocyte enlargement, and interstitial fibrosis. Apigenin also reduced oxidative stress by decreasing 4-hydroxynonenal and MDA levels and restoring glutathione peroxidas (GPx) and SOD activities and suppressed cardiomyocyte apoptosis through the modulation of Bax, Bcl-2, and cleaved caspase-3. Apigenin inhibited IκBα phosphorylation, TNF-α production, and NF-κB/p65 nuclear translocation. The cardio-protective effects were further confirmed in high glucose-treated H9c2 cardiomyocytes (25 μM).

Chrysoeriol (**149**, 20 mg/kg, p.o., 45 ds), isolated from *Cardiospermum halicacabum*, improved hyperglycemia and diabetes-associated dyslipidemia in STZ-induced diabetic Wistar rats [135]. It markedly reduced plasma glucose (282.16 to 133.27 mg/dL) and glycosylated hemoglobin (13.02 to 6.52 mg/g of Hb) while restoring serum insulin concentrations (6.23 to 15.87 µU/mL). In addition, chrysoeriol normalized lipid metabolism by reducing total cholesterol, triglycerides, free fatty acids, phospholipids, low-density lipoprotein, very low-density lipoprotein, and the atherogenic index. These effects were associated with the suppression of HMG-CoA reductase activity and stimulation of lipoprotein lipase (LPL) and lecithin cholesterol acyltransferase (LCAT).

Cedrol (**179**, supplemented at 0.2%) significantly decreased the body-weight gain, weight of visceral fat pad, and adipocyte hypertrophy in an HFD mouse model, followed by improving hepatic steatosis, glucose intolerance, insulin resistance [139]. No mortality or treatment-related clinical sign was recorded over the 19-week feeding period, and the authors noted that the acute dermal LD_50_ of cedrol exceeds 5000 mg/kg and that no deaths occurred at a maximum tolerated oral dose above 4 g/kg. GC-MS measurement showed accumulation of cedrol to 0.571 ± 0.143 μM in serum and 0.749 ± 0.116 μmol/kg in white adipose tissue.

*β*-sitosterol (**209**, 50 mg/kg, p.o., 30 ds) was reported to control insulin sensitivity in adipose tissue by stimulating the mRNA and protein expression levels of the insulin receptor (IR), the β-arrestin-2/c-Src complex, and the phosphorylation of insulin receptor substrate-1 (IRS-1), thereby increasing glucose uptake and oxidation in HFD type-2 diabetic rats [144]. Furthermore, **209** reduced insulin resistance by decreasing serine phosphorylation of IRS-1.

In an HFD/STZ-induced diabetic nephropathy (DN) rat model, treatment of *R. glutinosa* water extract (250 and 500 mg/kg) reduced blood glucose levels and improved lipid profiles, renal and hepatic function, antioxidant capacity, and renal pathological damage [154]. It also attenuated inflammatory responses and decreased the mRNA and protein expression of toll-like receptor 4 (TLR4), myeloid differentiation factor 88 (MyD88), and NF-κB p65 [154]. In a diabetic mouse model, total glycosides from the leaves of *R. glutinosa* (DHY) reduced blood glucose and lipid levels and mitigated diabetic kidney injury [155]. DHY also modulated gut microbiota and serum metabolites and suppressed the TGF-β/Smad signaling pathway [155]. Consumption of steamed *R. glutinosa* roots (8 g, 8 w) showed anti-obesity effects by significantly reducing waist circumference and modulating the gut microbiota in a clinical study involving 12 overweight middle-aged women (40–65 years) [152].

### 3.2. Anti-Inflammatory Activity

Aucubin (**1**, 1–30 μM) attenuated TNF-α-induced inflammatory response in 3T3-L1 adipocytes in a dose-dependent manner by inhibiting the secretion of the pro-inflammatory adipokines MCP-1, PAI-1, and interleukin (IL)-6 (IC_50_ = 8.20, 9.94, and 6.85 μM, respectively) [91]. At 10 μM of aucubin, the mRNA expressions of the adipokines were reduced by 24.3–39.0%. The underlying mechanism of action was associated with the inhibition of ERK phosphorylation, prevention of IκBα degradation, and subsequent suppression of NF-κB. Aucubin (0–240 μM) suppressed inflammatory cytokine and reactive oxygen species (ROS) production in LPS-induced HepG2 cells [101]. It (25, 50, or 100 mg/kg) also mitigated the pathological symptoms of acute hepatitis in mice by signal transducers and activators of the transcription 3 (STAT3)/NF-κB signaling pathway.

Geniposide (**11**) exhibited anti-inflammatory activity in a collagen-induced arthritis C57BL/6 mice model [105]. Intraperitoneal administration of geniposide (60 and 120 mg/kg/d for 14 ds) significantly suppressed the production of pro-inflammatory factors [IL-17, TNF-α, IL-8, MMP-3, and MMP-9) while increasing IL-10 levels. Histological analysis further showed reduced inflammatory-cell infiltration and cartilage destruction. In TNF-α-stimulated RA-FLS cells, geniposide inhibited inflammatory mediator production by suppressing JAK1 and STAT1 phosphorylation. Furthermore, geniposide (200, 400, and 500 mg/kg/d for 7 ds) exhibited anti-inflammatory activity in STZ-induced diabetic rats by accelerating lesion regression by 1.06–1.84 times [106]. Treatment with compound 11 accelerated wound closure, attenuated inflammatory cell infiltration, and promoted fibroblast proliferation in the lesion area. These effects were accompanied by reductions in TNF-α, IL-1β, and IL-6 levels and a dose-dependent increase in IL-10 expression. Geniposide was cytotoxic to normal human fibroblast-like synoviocytes at 200 μM after 24 and 48 h but had no effect on their proliferation at 0–100 μM, which is why 25–100 μM was used throughout the cell work; the authors note, as a limitation, that organ toxicity was not assessed and that the pharmacokinetics of geniposide in a physiological context remain to be established.

Rehmaglutin B (**53**) and isoacteoside (**123**) suppressed NO production in LPS/IFN-γ- and LPS-treated RAW 264.7 cells without cytotoxicity (IC_50_ = 52.8 ± 2.0 and 44.6 ± 4.2 µM, respectively). Rehmaglutin B was more potent than the positive control N^G^-monomethyl-L-arginine (L-NMMA), whereas isoacteoside was less potent than L-NMMA (IC_50_ = 22.1 ± 0.1 µM) [117,123]. Rehmaglutin D (**54**) reduced NO production in LPS-treated BV2 microglial cells with IC_50_ value of 53.1 ± 2.7 µM (IC_50_ of butein, a positive control = 4.5 ± 0.3 µM) [118]. Interestingly, the active component responsible for the anti-inflammatory activity of luteolin (**148**) was determined to be luteolin glucuronides, especially luteolin-3′-*O*-glucuronide, in the in vivo mouse model (20 mg/kg) [134]. After a single oral dose of 20 mg/kg to rats, luteolin-3′-*O*-glucuronide reached a peak plasma concentration of 4.1 μM at 3 h while free luteolin was barely detectable, and 25 μM of the aglycone reduced RAW264.7 viability to 65% of control; the concentrations at which luteolin itself is active in vitro are therefore both cytotoxic and above what oral dosing achieves in plasma, whereas the glucuronides were not cytotoxic up to 50 μM.

Rehmapicrogenin (**173**, 25 and 50 mg/kg) treatment significantly improved serum creatinine (Scr), blood urea nitrogen (BUN), and urine albumin (U-ALB) in a mouse model of doxorubicin (DOX)-induced kidney failure [136]. It also improved NRK-52E cell viability (a rat kidney tubular epithelial cell line) and the protein levels of Nrf2, KEAP1, HO-1, and HDAC2 in NRK-52E cells. In addition, administration of cedrol (**179**, 7.5, 15, and 30 mg/kg) decreased the levels of TNF-α, MDA, and urea and improved the thiol content in an LPS-exposed rat model, with the strongest effects observed at 30 mg/kg [140]. Rehmapicrogenin reversed the model-induced loss of body weight (17.28 ± 0.58 g in the model group against 18.38 ± 0.41 g at 25 mg/kg and 18.30 ± 0.45 g at 50 mg/kg, with 19.36 ± 0.23 g in normal controls) and was not cytotoxic to NRK-52E cells at 1–10 μmol/L.

Topical application of steamed *R. glutinosa* roots (400 μg/mouse) significantly improved dermatitis scores, ear thicknesses, and serum histamine levels [156]. It also decreased inflammatory cell infiltration and suppressed inflammatory cytokines (IL-4 and TNF-α), adhesion molecules (VCAM-1 and ICAM-1), and chemokines (TARC, MDC, and RANTES) in the ear lesions and keratinocytes [156].

### 3.3. Neuroprotective Activity

Aucubin (**1**, 50, 100, or 200 μg/mL) produced neuroprotective effects in vitro in H_2_O_2_-injured primary mouse cortical neurons, and also it (20 and 40 mg/kg) significantly relieved brain edema, histological damages, and cognitive deficits in a traumatic brain injury mouse model [102]. Treatment of catalpol (**43**, 5.0 mg/kg, i.p. injection at 12, 24, 48, and 72 h) stimulated GSH-PX activity (14.2 ± 6.0 U/mg prot in cortex and 15.5 ± 4.6 U/mg prot in hippocampus) and suppressed the production of MDA (4.2 ± 2.0 nmol/mg prot and 4.4 ± 1.9 nmol/mg prot), thus resulting in decreased apoptosis, attenuated neuronal damage in the hippocampal CA1 region, and improved cognitive impairment [158]. Intraperitoneal administration of rehmannioside A (**45**, 80 mg/kg) helped to decrease the region of cerebral infarction in the middle cerebral artery occlusion (MCAO) mouse model by stimulating PI3K/AKT/Nrf2 and SLC7A11 pathways [159].

Phenylethanoid glycosides, isoacteoside (**123**), acteoside (**124**), echinacoside (**125**), and 2′-acetylacteoside (**131**), isolated from *Callicarpa dichotoma*, showed neuroprotective effects in glutamate-injured rat cortical cells (MK-801 as a positive control), increasing cell viability by 67.9%, 62.9%, 68.3%, and 73.5% at 10 µM, respectively [124].

Rehmapicroside (**174**, 2.5, 5, and 10 mg/kg) was reported to suppress 3-nitrotyrosine formation, Drp1 nitration, NADPH oxidases, and iNOS expression in the MCAO rat model by suppressing ONOO^−^-mediated mitophagy signaling pathway [138]. It also inhibited the translocations of key proteins related to mitophagy activation—PINK1, Parkin, and Drp1—reduced infarct sizes, and improved neurological deficit scores. Rehmapicroside was not cytotoxic to PC12 cells over 0.39–100 μM for 24 h, and the authors attributed its failure to reduce mitochondrial Drp1 nitration at 10 mg/kg to insufficient bioavailability at the mitochondrion. Furthermore, cedrol (**179**, 40 mg/kg) showed neuroprotective effects against acute cerebral ischemia by suppressing microglia-associated inflammation via ERβ-NF-κB signaling pathways in the MCAO mouse model [141]. Cedrol did not affect microglial viability between 5 and 50 μM but reduced it concentration-dependently between 100 and 250 μM, so all cell work was confined to the lower range. The treatment of daucosterol (**210**, 10 and 20 μM) improved neuronal loss, apoptotic rate, and caspase-3 activity by stimulating insulin-like growth factor I (IGF1), which shows anti-apoptotic activities in neurons [145]. 5-Hydroxymethyl furfural (**235**) increased the protein levels of the glucocorticoid receptor (GCR), brain-derived neurotrophic factor (BDNF), N-methyl-D-aspartate receptor 2B (NR2B), cAMP response element binding protein (p-CREB), phosphorylation-extracellular signal-regulated kinase (p-ERK), and phosphorylation-synapsin (p-synapsin) and reduced the activity of *β*-galactosidase [148].

### 3.4. Other Organ-Protective Activities

Aucubin (**1**) has been reported to show beneficial effects in nonylphenol(NP)-induced testicular damage in male rats (male albino rats 190–210 g, 5 mg/kg) [103]. Specifically, the compound restored the activities of antioxidant enzymes (GSR, CAT, SOD, and GPx) and total protein content (TPC) while reducing the levels of ROS and thiobarbituric acid-reactive substances (TBARS) and the hormone levels of FSH, LH, and testosterone. It increased the number, motility, and membrane integrity of spermatocytes and also corrected the sperm abnormalities and the number of nonviable sperm. In addition, it reduced levels of the pro-apoptotic proteins, including Bcl-2 associated X protein (Bax), caspase-9, and caspase-3, which were released by NP treatment. Aucubin given alone at 5 mg/kg for 56 ds did not differ from untreated controls in any of the antioxidant, hormonal, steroidogenic, apoptotic, or sperm parameters measured, indicating that the compound was without intrinsic effect at the protective dose; no independent dose-ranging or toxicity study was performed, the dose having been adopted from an earlier report.

Catalpol (**43**) has been reported to exert multiple organ-protective effects. For example, rats with low and high dosages of catalpol (5 and 10 mg/kg/d, respectively) showed protection against the isoproterenol-induced cardiac dysfunction [113]. It also increased the level of apelin, an endogenous peptide that plays a protective role in the cardiovascular system, and inhibited cardiomyocyte apoptosis. Catalpol (**43**) has also demonstrated neuroprotective activity. To be specific, it activated STAT3 and increased VEGF production, leading to increased angiogenesis and improved behavioral outcomes after stroke in a rat model (5 mg/kg, i.p.) [114]. Moreover, treatment of catalpol (2.5, 5, and 10 mg/kg) decreased the lung wet-to-dry (W/D) ratio and the levels of inflammatory cytokines—TNF-α, IL-6, IL-4, and IL-1β—in mice in an LPS-induced acute lung injury model [115]. The radio-protective activity of catalpol (25–100 μg/mL) was demonstrated in vitro by the inhibition of apoptosis in ionizing radiation (IR)-induced human lymphocytes AHH-1 cells [116]. In 8 Gy-irradiated male BALB/c mice treated with catalpol (25, 50, and 100 mg/kg, i.p.), the morphological damage of the gastrointestinal tract deceased by 15.6%, 33.3%, and 44.4%, respectively, and several plasma levels, including endogenous antioxidants, peripheral white blood cells, and platelets, were increased.

Monomelittoside (**3**), rehmaglutoside L (**5**), (8*S*)-7,8-dihydrogeniposide (**31**), and mussaenoside (**33**) (50 μM each) showed protective activities in rat H9c2 cardiomyocytes treated with 50 μM DOX [10]. Furthermore, salidroside (**114**) significantly alleviated high mortality, cardiac malfunction, and increased infarct size in C57BL/6 J male mice (myocardial infarction model, 200 mg/kg/d) [122]. It also improved myocardial inflammation and apoptosis and decreased the expression levels of TNF-α, TGF-β1, IL-1β, and Bax. Compounds frehmaglutoside I (**76**) and rhemaneolignans A (**163**) and B (**164**) (40 and 80 μM) showed cardio-protective activities against DOX-induced cardiotoxicity in rat cardiac H9c2 myocardial cells [33]. Geniposide (**11**) significantly attenuated cardiac hypertrophy or dysfunction induced by transverse aortic constriction or isoprenaline treatment in a mouse model (25 or 50 mg/kg) through activation of the GLP-1 receptor/AMPK-α signaling pathway [107]. Geniposide (10–40 mg/kg, p.o., four w) produced no adverse effect in that model. Furthermore, Yao et al. reported that compound **11** strongly improved cardiac hypertrophy, ventricular remodeling, myocardial fibrosis, and cardiac function in a transverse aortic contraction-induction mice model (male C57BL/6 mice, 0, 10, 20, and 40 mg/kg, p.o.) [108]. The underlying mechanism of actions was revealed as the transforming growth factor β1 (TGF-β1)/Smad2 pathway. Angiotensin converting enzyme (ACE) plays a key role in the renin–angiotensin system (RAS), a major regulatory system in cardiovascular physiology. ACE converts angiotensin I into the potent vasoconstrictor, angiotensin II, and promotes the degradation of bradykinin, a vasodilatory peptide. Thus, excessive ACE activity may contribute to increased blood pressure. Phenylpropanoid glycosides (**124**, **128**, **133**, and **135**) from *Clerodendron trichotomum* showed significant inhibition of rat plasma ACE in a dose-dependent manner, and the IC_50_ values were 373 ± 9.3 µg/mL, 423 ± 18.8 µg/mL, 505 ± 26.7 µg/mL, and 524 ± 28.1 µg/mL, respectively [126]. In a carrageenan-induced thrombosis rat model, antithrombotic effect of the Rehmanniae Radix water decoction (1.35, 2.7, and 5.4 g/kg) was observed, and the integrated metabolomic and network pharmacology approaches revealed that these effects may involve the regulation of PLA2G2A, PTGS1, ALOX5, and CYP2C9 [153].

Rehmannioside D (**6**, 10 and 40 mg/kg) exerted protective effects against liver fibrosis in a CCl_4_-treated mice and dimethylnitrosamine (DMN)-exposed rats by suppressing the Sirt/p53 signaling pathway and hepatocyte apoptosis [104]. Rehmaglutoside C (**26**), rehmaglutoside J (**47**), rehmaglutoside K (**48**), frehmaglutin C (**83**), rehmaglutoside I (**107**), and rehmaglutoside G (**109**) (10 μM each) showed mild hepatoprotective effects against D-galactosamine-induced cell damage, with inhibition rates ranging from 24.4% to 38.7% [22,31,127]. Acteoside (**124**) isolated from *Leonurus japonicus* showed comparable hepatoprotective activity in the D-galactosamine/HL-7702 assay. In addition, the tested iridoid glycosides showed no cytotoxic activity against five human tumor cell lines (HCT-8, Bel-7402, BGC-823, A549, and A2780; IC_50_ > 10 μM). Moreover, compounds ajugol (**13**), catalpol (**43**), and rehmaglutoside F (**104**) (10 μM each), isolated from *R. chingii*, exhibited hepatoprotective activities in APAP-injured HepG2 cell damage (bicyclol 10 μM as the positive control) [109]. Radix Rehmanniae Praeparata (steamed roots of *R. glutinosa*) and its fractions alleviated hepatic inflammation and sinusoidal injury [157]. RRP glucosides (RRP_GLY_) attenuated senescence in liver sinusoidal endothelial cells and suppressed pro-inflammatory senescence-associated secretory phenotype genes through inhibition of the LRP1–NOTCH1–C/EBPβ signaling pathway in a hepatic ischemia-reperfusion injury mouse model [157].

Liu et al. reported that frehmaglutin D (**101**, 10 mg/kg) and rehmaionoside C (**88**, 10 mg/kg) relieved the pathological symptoms of sepsis-induced acute kidney injury (S-AKI) in the LPS-induced S-AKI mouse model by reducing renal damage, inflammation, uncontrollable immune cells, and ROS levels [120]. The protein expressions of ERα and ERβ were increased, whereas those of TLR4, caspase 11, and IL-1β were decreased. 11-Methylforsythide (**32)** exhibited reno-protective activity against LPS-treated rat kidney tubule epithelioid (EC_50_ = 45.1 μM) [17]. *sec*-Hydroxyaeginetic acid (**71**), dihydroxy-*β*-ionone (**84**), trihydroxy-β-ionone (**90**), and frehmaglutin D (**101**) showed moderate protective effects against LPS-induced normal rat kidney tubule epithelioid (NRK-52E) cells (EC_50_ = 10.2–40.7 µM) [62]. Combination therapy of acteoside (**124**, 0.8 g/d) and valsartan(80–160 mg/d) was conducted in a relatively small cohort comprising 31 patients with IgA nephropathy and 16 healthy controls, and the results showed that the treatment improved the proteinuria and Th22 lymphocytosis of patients [125]. Acteoside (**124**) also suppressed the production of chemokines (CCL20, CCL22, and CCL27) and pro-inflammatory cytokines (IL-1, IL-6, and TNF-α) in the Th22 cells [125]. Nepetoidin F (**145**), nicotinic acid (**271**), and rehmagluamide (**304**) (10 μM each) also showed reno-protective effects against the LPS-induced damage to NRK-52E cells [49]. Remophilanetriol A (**188**) and remophilanetriol B (**190**) (10 μM each) also showed protective activities against LPS-induced damage in NRK-52E rat kidney cells [45].

2,5-Dihydroxyacetophenone (**252**) improved the survival rate and reduced the serum lactate dehydrogenase activity and serum lactate level in a cecal ligation and puncture-induced sepsis-associated acute kidney injury rat model (40 or 80 mg/kg, p.o.) [150]. It also suppressed the release of TNF-α, IL-1β, and TGF-β1, serum creatinine, and kidney injury molecule-1 through inhibiting ERK and NF-κB signaling pathways. Oleic acid (**313**) inhibited inflammation and oxidative stress through the Ras/MAPKs/PPAR-γ signaling pathway, phenylalanine metabolism, purine metabolism, sphingolipid metabolism, and taurine/hypotaurine metabolism in an LPS-induced acute kidney mouse model [151].

Mussaenoside (**33**), 8-epiloganin (**35**), gardoside methyl ester (**37**), and remophilanetriol F (**187**) (10 μM) showed protective effects in TGF-β1-stimulated BEAS-2B bronchial epithelial cells, an in vitro model of pulmonary fibrosis. In addition, compound **187** decreased the levels of ROS and apoptosis in TGF-β1-induced BEAS-2B cells [19]. Frehmaglutin B (**64**), frehmaglutin B_1_ (**65**), aeginetic acid 5-*O*-*β*-D-quinovoside (**66**), frehmaglutin A (**73**), rehmaglutin A_1_ (**74**), *sec*-hydroxyaeginetic acid lactate (**79**), rehmaionoside C (**88**), abscisic acid (**96**), rehmapicrogenin (**173**), and rehmapicrogenin monomethyl ester (**178**) (10 μM) showed strong anti-pulmonary fibrosis activity [61]. In addition, frehmaglutin B_1_ (**65**) exhibited protective activity against TGF-β1-induced BEAS-2B injury through the PI3K/AKT/NF-κB signaling pathway (EC_50_ = 10.4 μM). Jiofuran acid A (**146**), (+)-paulownin (**156**), and 8′-hydroxyl episesaminone (**157**) (10 μM) showed strong anti-pulmonary fibrosis activity in TGF-β_1_-induced BEAS-2B cells [66].

Interestingly, the hair regenerative activity of cedrol (**179**) was also investigated [142]. It (30 mg/kg) controlled the M1/M2 polarization imbalance by utilizing the STAT and MAPK signaling pathways in alopecia areata mouse model. The pro-osteogenic effects of ajugoside (**14**, 100, 200, and 400 mg/kg, p.o.) was supported by the activation of osteogenic differentiation through Slc5a1 inhibition and AMPK-mediated signaling pathways in an ovariectomy-induced osteoporosis mouse model (female C57BL/6J mice) [111]. Ajugoside had no effect on bone marrow stromal cell viability at 1–100 μM but reduced it at 150 and 200 μM; in the ovariectomized mice, general condition and body weight were monitored throughout, and no abnormality was observed, although the authors state that long-term safety and pharmacokinetic data for ajugoside are lacking and must be obtained before clinical translation. Furthermore, 5-HMF (**235**, 7.5–30 mg/kg) was reported to have protective effects on osteoarthritis by regulating the Glut1/HK1/LDHA signaling pathway in the destabilization of Medial Meniscus (DMM)-induced osteoarthritic mice [149].

### 3.5. Cytotoxic and Antitumor Activities

A clinical study involving 345 patients who underwent surgical resection of locally advanced colon cancer was conducted to evaluate the anti-cancer effects of catalpol (**43**, 10 mg/kg, i.p., twice per day, 12 w) [112]. Treatment of catalpol significantly reduced the serum levels of carbohydrate antigen 19-9 (CA 19-9), carcinoembryonic antigen (CEA), and matrix metalloproteinases (MMP)-2 and MMP-9. Furthermore, patients receiving catalpol treatment showed improved overall survival (OS) and cancer-free survival (CFS) compared with the placebo group.

Jionoside C (**113**) isolated from the leaves of *Clerodendrum infortunatum* (Lamiaceae) showed mild cytotoxic activity against Hs578T cells (IC_50_ = 85.3 ± 2.4 μM) and MDA-MB-231 cells (IC_50_ = 96.5 ± 1.5 μM), which are triple-negative breast cancer cell lines [121]. Administration of acteoside (**124**, 50 mg/kg) significantly extended the survival time (63.3 ± 3.4 d) of mice in a B16 lung metastasis model compared to the control (52.1 ± 2.5 d) [128]. In addition, acteoside (**124**, 10^−6^–10^−8^ M) and martynoside (**135**, 10^−6^–10^−8^ M) were reported to exert strong anti-estrogenic and antiproliferative effects in MCF-7 cells [129].

Cedrol (**179**, 20–30 μg/mL), isolated from *Juniperus chinensis*, reduced the expression level of minichromosome maintenance (MCM) proteins associated with G1 arrest and apoptosis in human colorectal cancer HT29 cells [143]. In addition, administration of cedrol (50 mg/kg) significantly inhibited tumor growth without weight loss and vascularization in a human tumor xenograft model. Cedrol-treated mice maintained a stable body weight throughout the 14 d treatment period, whereas DOX at 3 mg/kg caused body-weight loss. Although the in vivo findings provide stronger evidence than cell-based cytotoxicity alone, further toxicological and pharmacokinetic evaluation is required.

Glutinosalactone C (**208**), a new ursane-type triterpenoid isolated from *R. glutinosa*, exhibited cytotoxicity against three human cancer cell lines: MCF-7 (IC_50_ = 8.35 ± 0.16 μM), MG63 (IC_50_ = 39.25 ± 0.21 μM), and HepG2 (IC_50_ = 17.29 ± 2.16 μM), respectively [46]. Among these cell lines, the greatest potency was observed against MCF-7 cells.

In 2015, Zhao et al. reported the anti-proliferative effects of daucosterol (**210**), showing IC_50_ values of 19.96 μM for MCG803, 3.13 μM for BGC823, 24.19 μM for AGS, and 16.95 μM for MCF-7, respectively [146]. Furthermore, daucosterol (0.5 and 2.5 mg/kg, p.o., 7 ds) inhibited H22 hepatoma growth in an allograft mouse model, reducing tumor weight from 1.51 ± 0.23 g (control group) to 0.71 ± 0.29 (daucosterol 0.5 mg/kg) and 0.46 ± 0.28 g (daucosterol 2.5 mg/kg), respectively. Neither dose caused body-weight loss, and macroscopic examination found no lesions in the heart, liver, kidney, or lung. These in vivo findings strengthen the evidence for antitumor activity; however, assessment of cancer-cell selectivity, comparison with established anticancer agents, and further safety evaluation are necessary to determine its therapeutic relevance.

Rehmanniaeketone C (**242**), rehmanniaeketone B (**244**), and 3,8-dihydroxy-2-(2-hydroxyethyl)chromone (**264**) isolated from the roots of *R. glutinosa* showed anti-proliferative activities against human lung carcinoma A549 cells with the IC_50_ values of 2.1, 5.2, and 61.0 μM, respectively [74]. Rehmanniaeketones B and C therefore exhibited substantially greater in vitro potency than compound **264**. Nevertheless, these results demonstrate antiproliferative activity in a single cancer cell line, and their selectivity and therapeutic relevance remain uncertain in the absence of comparisons with appropriate reference drugs and in vivo validation.

### 3.6. Antioxidant Activity

As a Nrf2 agonist, jiofuran (**59**, 25 μM) showed antioxidant activity by activating the Nrf2 signaling pathway in 293 T cells [119]. Jionoside A_1_ (**129**, 0.1–50 μM) showed immune-enhancement activity in BALB/C mice bone marrow cells in a dose-dependent manner, and isoacteoside (**123**) and acteoside (**124**) demonstrated antioxidant activities on H_2_O_2_-treated SH-SY5Y cells (10 and 50 μM) [63].

Jionoside D (**134**, 0, 0.1, 1, and 10 μg/mL) showed significant scavenging activity of intracellular ROS (85% at 10 μg/mL) and the 1,1-diphenyl-2-picrylhydrazyl (DPPH) radical (55% at 10 μg/mL) [130]. It increased the cell viability of lung fibroblast cells by 11% at 0.1 μg/mL, 34% at 1 μg/mL, and 70% at 10 μg/mL.

Rehmapicrogenin (**173**, 5 and 10 μM) showed protective activity against LPS-induced podocyte apoptosis, and **173** (25 and 50 mg/kg) improved renal function in LPS-induced kidney dysfunction mouse model by activating the Nrf2/ARE signaling pathway [137].

### 3.7. Antimicrobial Activity

Co-administration of aucubin (**1**, 100 to 1000 μM) with *β*-glucosidase inhibited DNA replication of hepatitis B virus (HBV). In contrast, aucubin mono-treatment showed minimal antiviral activity, resulting in a 3.0-fold reduction in extracellular HBV virion DNA at 1000 μM [100]. The minimum inhibitory concentrations (MICs) of ajugol (**13**) were 31.25 μg/mL against *Bacillus subtilis*, *Staphylococcus aureus*, and *Escherichia coli* and 62.5 μg/mL against *Pseudomonas aeruginosa*, *Candida albicans*, and *Cryptococcus neoformans* [110]. Mice treated with daucosterol (**210**, 40 µg twice on a 3 d interval) prolonged the survival of mice with disseminated candidiasis through a CD4+ Th1 immune response, thus producing IFN*γ* and IL-2 cytokines [147].

## 4. Conclusions

In this review, the phytochemical constituents from *R. glutinosa* were systematically reviewed and classified according to their structural characteristics, including iridoids, ionones, phenyl ethanoids, phenyl propanoids, flavonoids, lignans, terpenoids, alkaloids, and saccharides. Furthermore, the reported biological activities of *R. glutisosa* and its compounds were summarized: antidiabetic, anti-inflammatory, organ-protective, cytotoxic, antioxidant, and antimicrobial activities. This review presents a comprehensive overview of the phytochemical profile of the plant and its associated biological activities, providing a foundation for further investigation of the potential therapeutic applications.

## Figures and Tables

**Figure 1:**
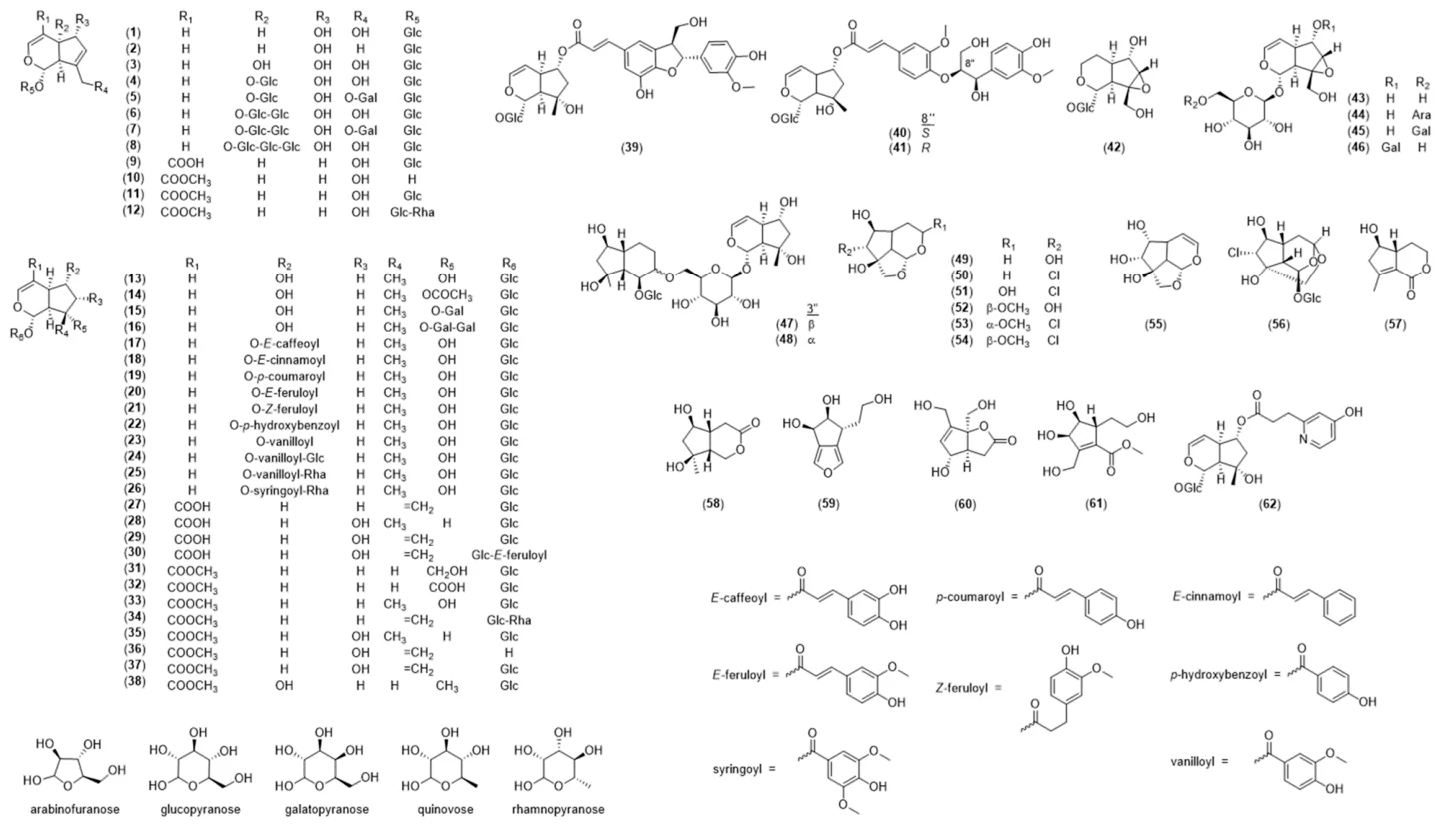
Iridoids and their glycosides from *R. glutinosa.*

**Figure 2:**
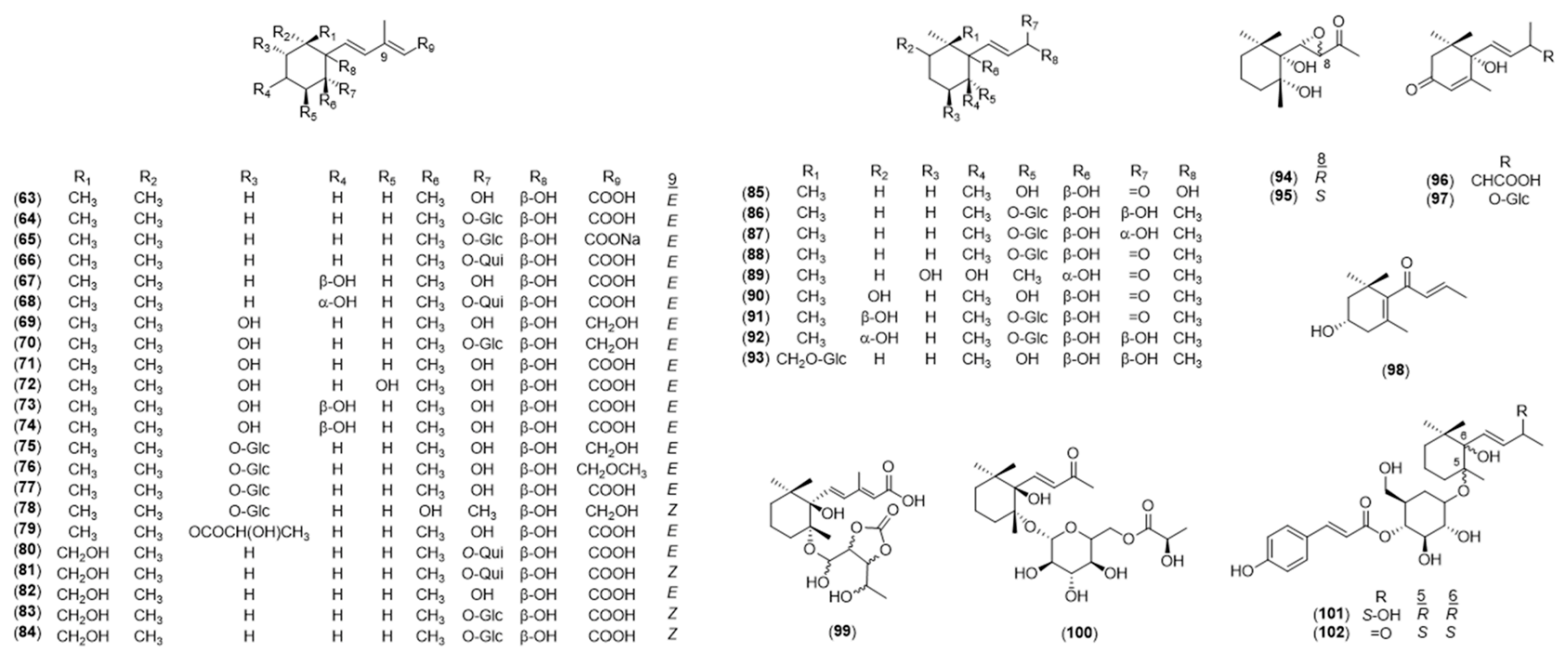
Ionones and their glycosides from *R. glutinosa.*

**Figure 3:**
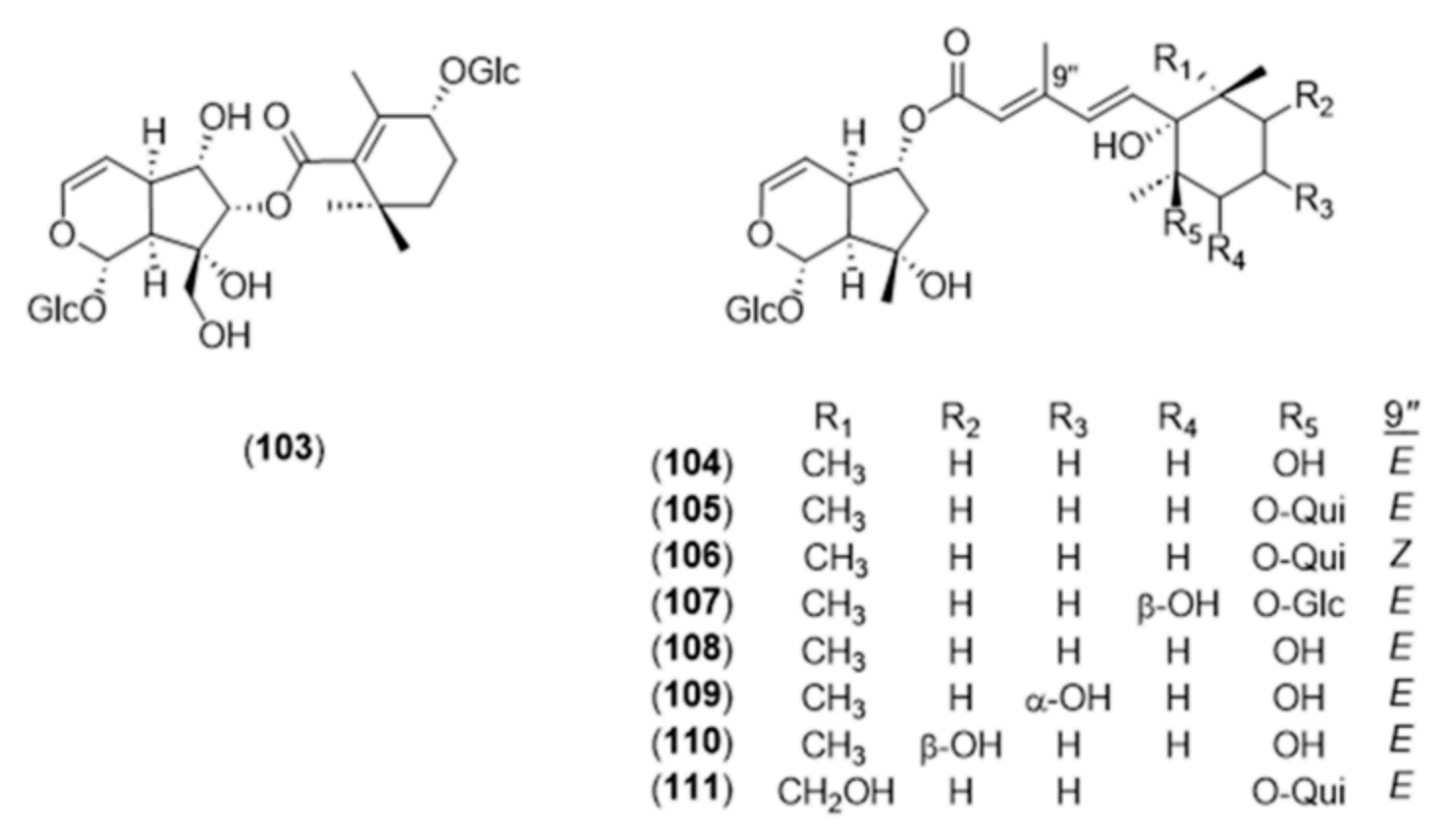
Glycosides of iridoid–ionone conjugates from *R. glutinosa.*

**Figure 4:**
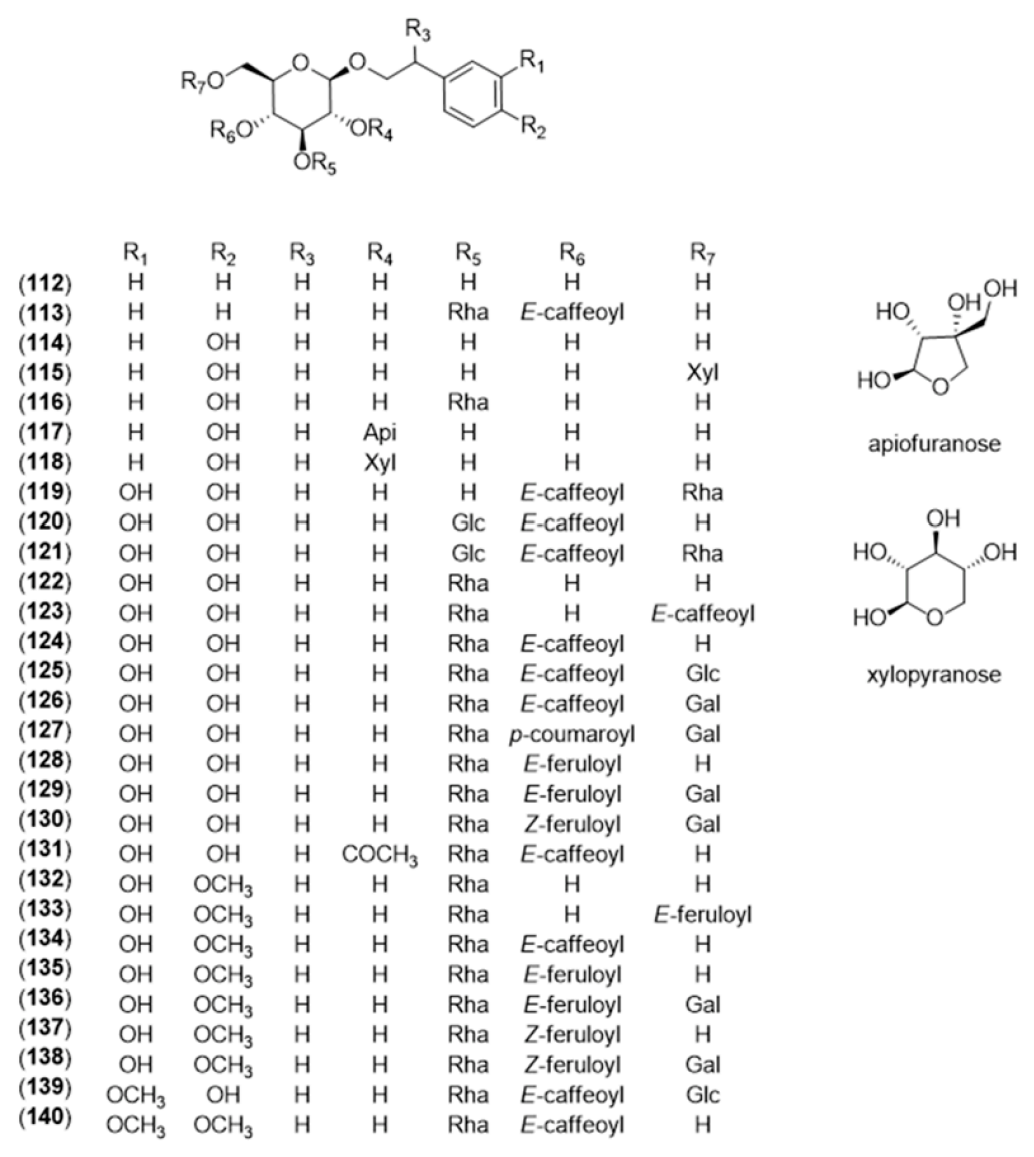
Phenylethanoid glycosides from *R. glutinosa.*

**Figure 5:**
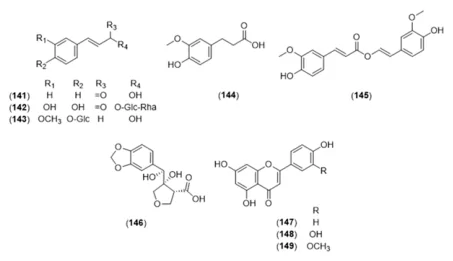
Phenylpropanoids, phenylpropanoid glycosides, and flavonoids from *R. glutinosa.*

**Figure 6:**
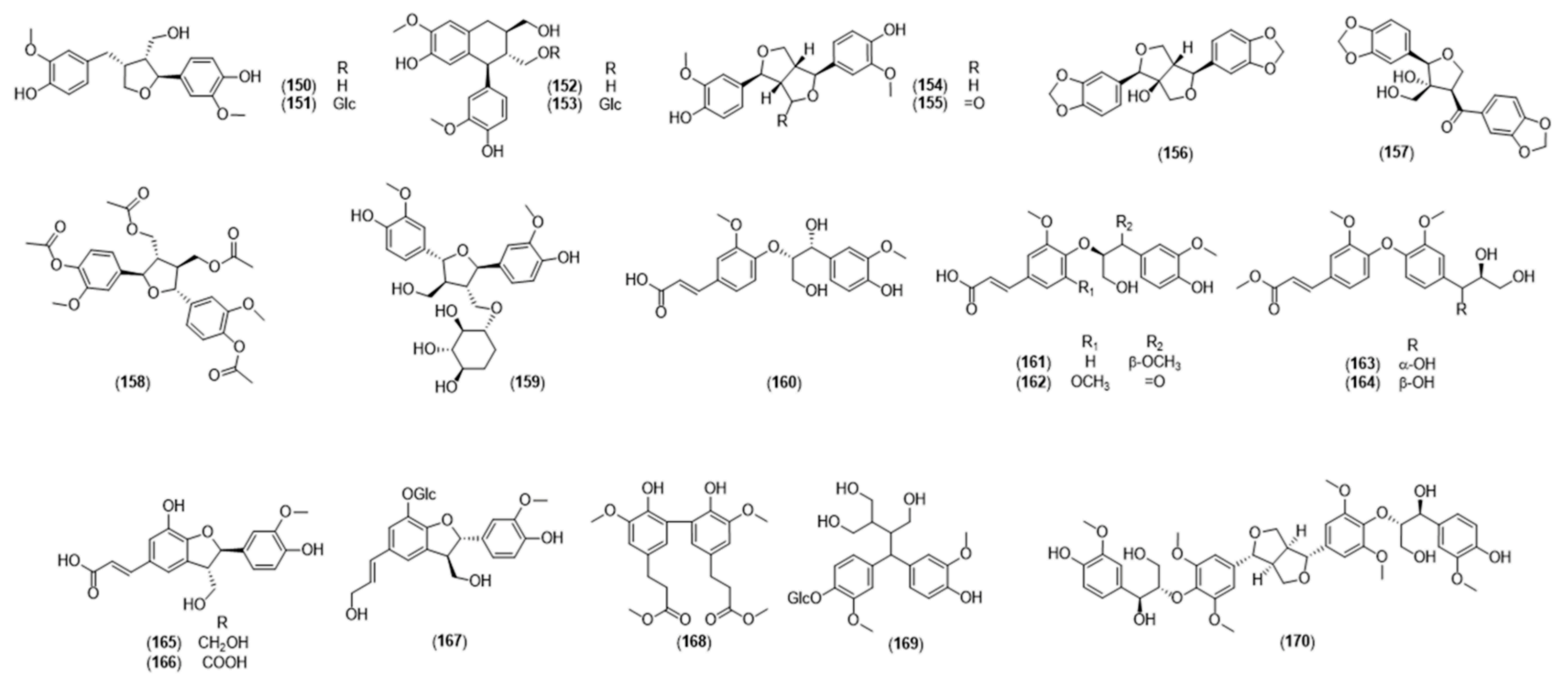
Lignans and their glycosides from *R. glutinosa.*

**Figure 7:**
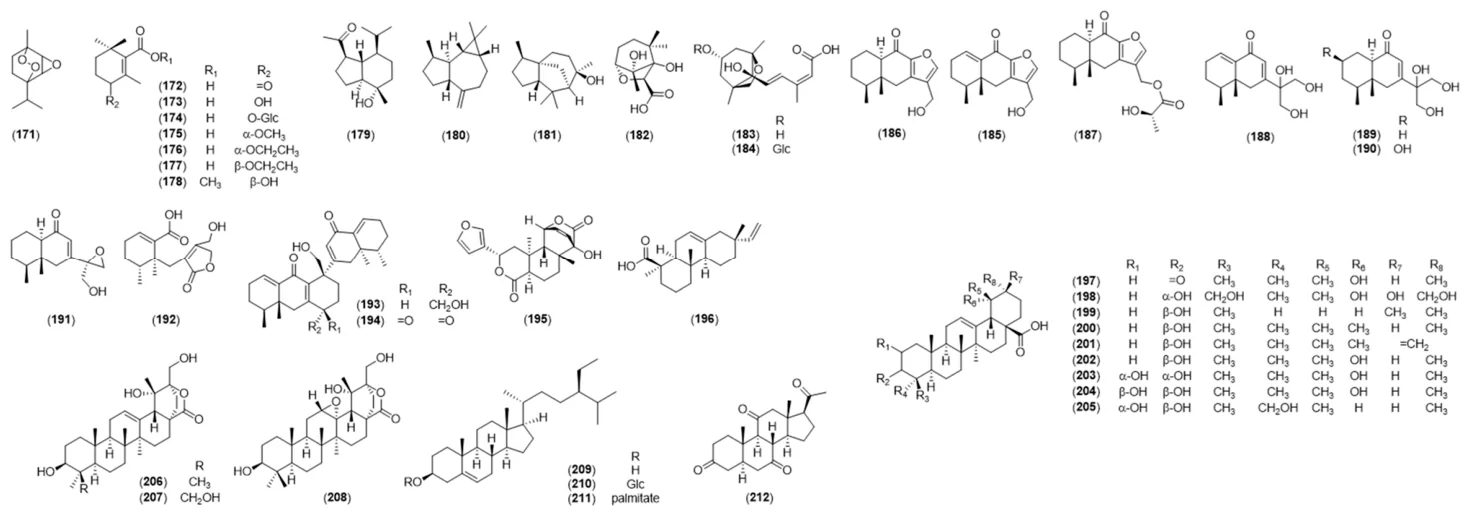
Terpenoids isolated from *R. glutinosa.*

**Figure 8:**
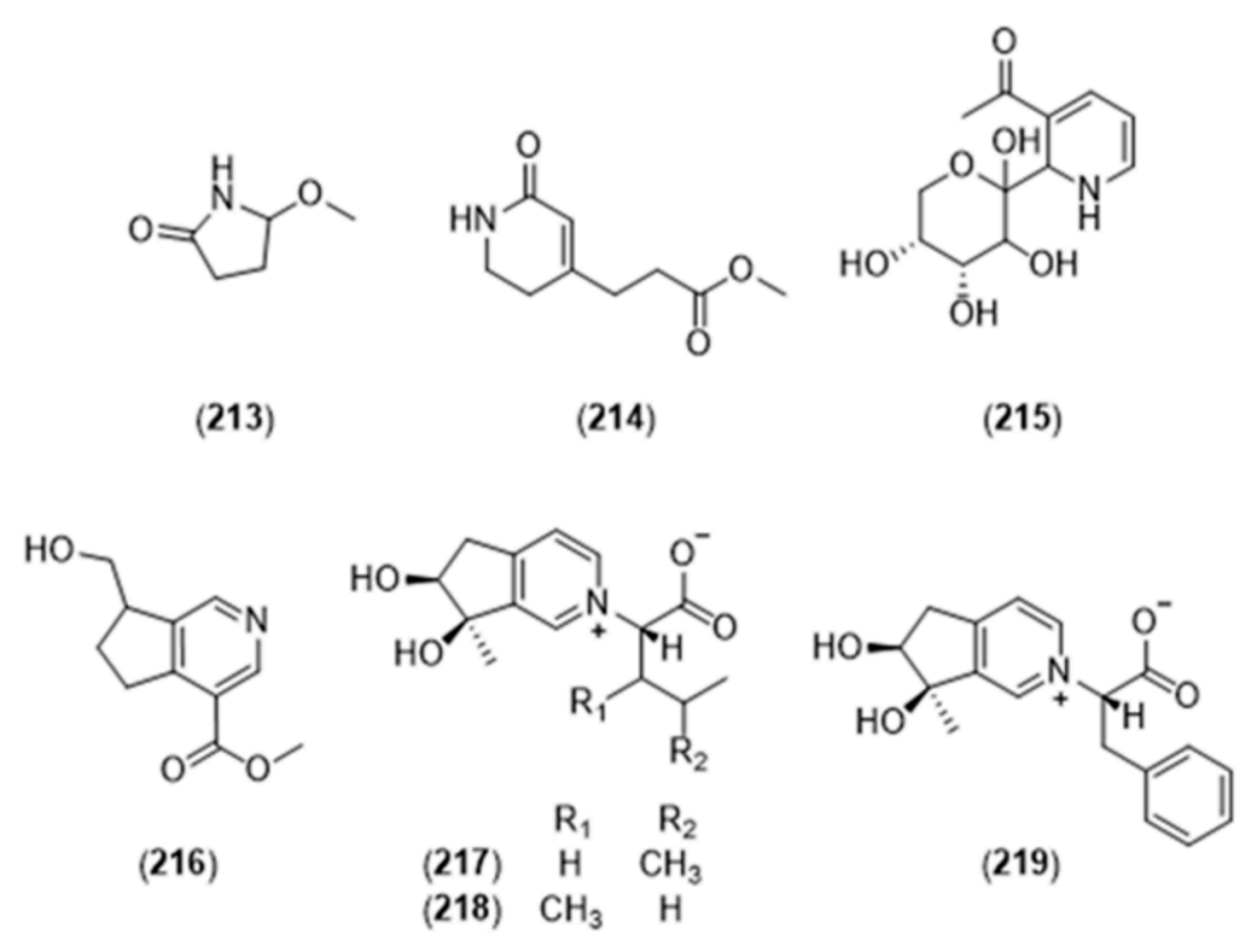
Alkaloids from *R. glutinosa.*

**Figure 9:**
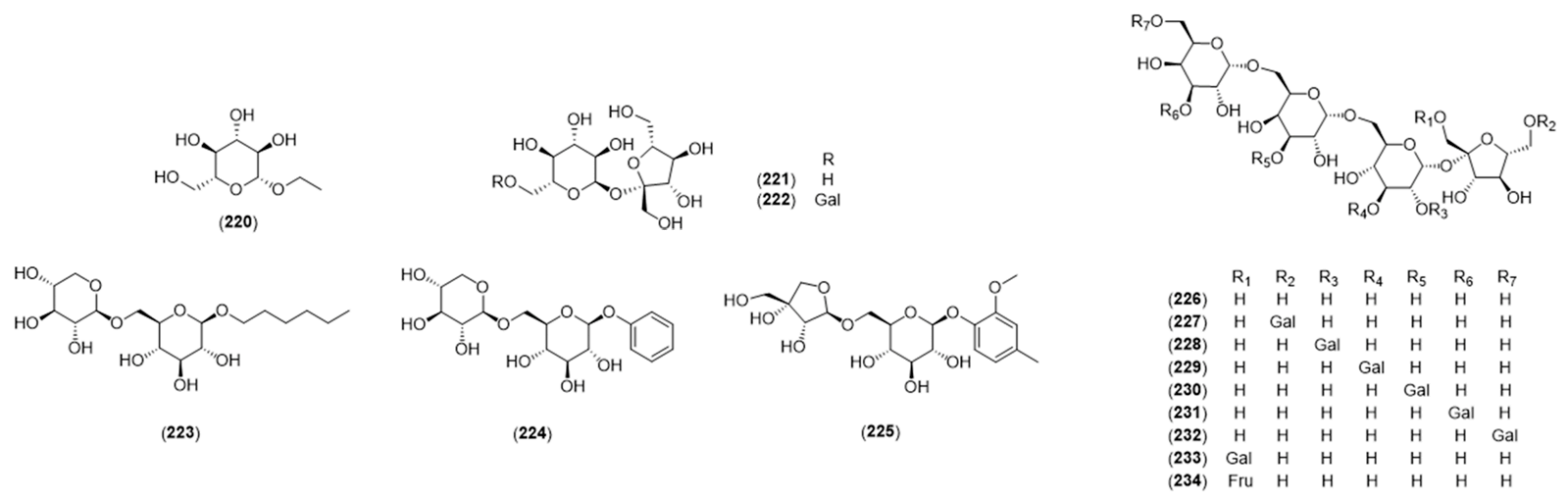
Saccharides from *R. glutinosa.*

**Figure 10:**
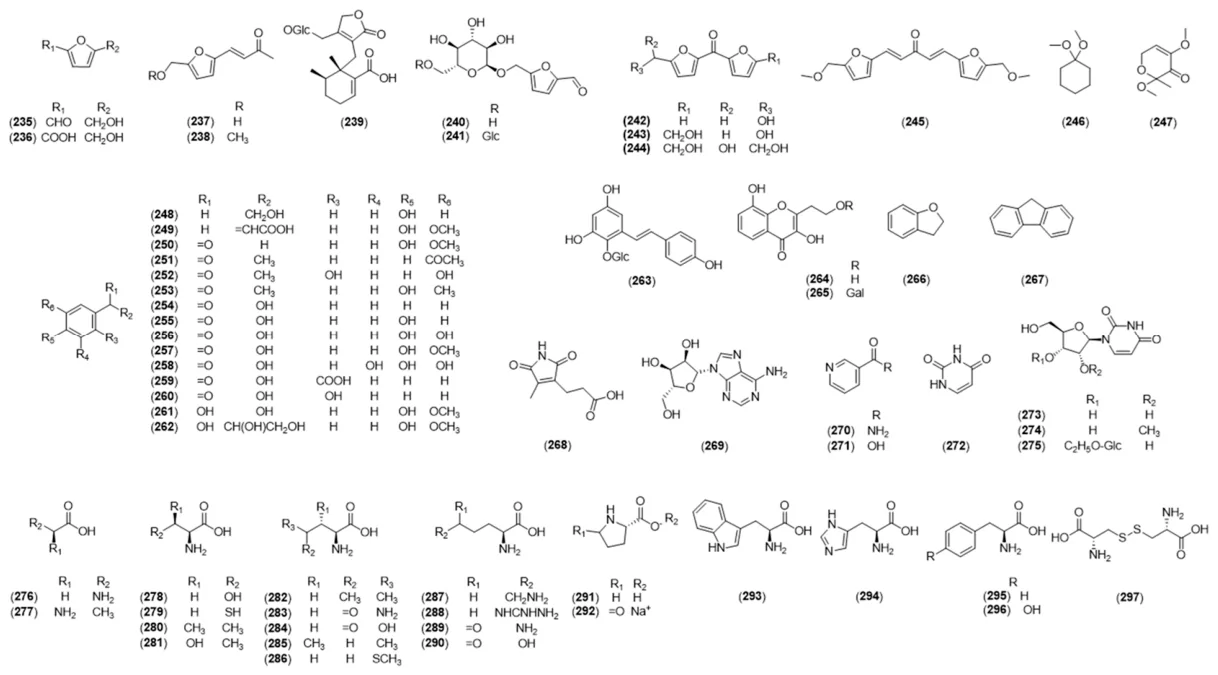
Other compounds from *R. glutinosa.*

**Figure 11:**
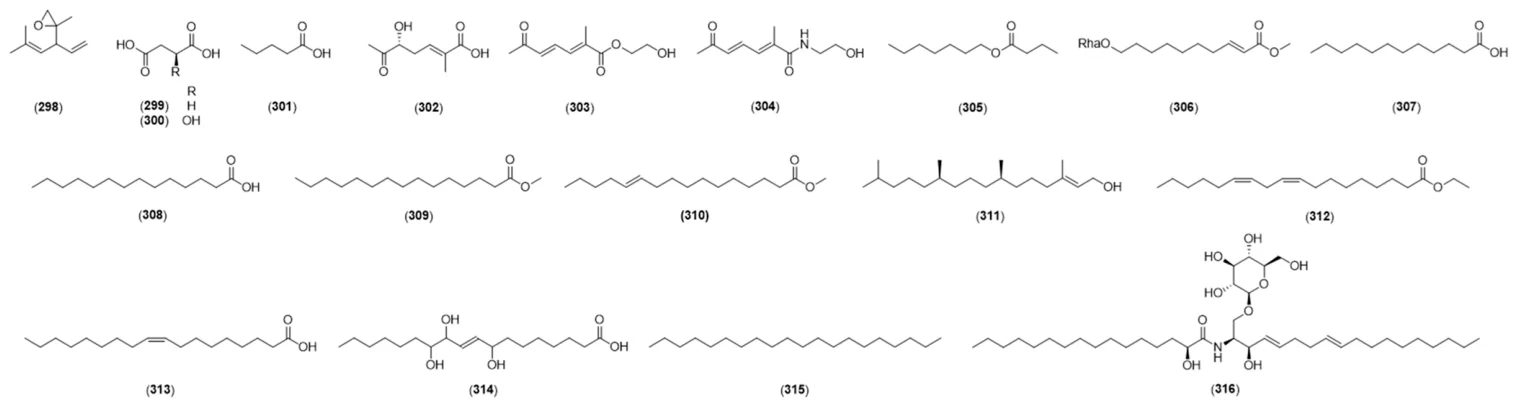
Miscellaneous compounds from *R. glutinosa*.

## Data Availability

The original contributions presented in this study are included in the article. Further inquiries can be directed to the corresponding authors.
